# Servant versus directive leadership and promotability: does leader gender matter?

**DOI:** 10.3389/fpsyg.2023.957121

**Published:** 2023-12-11

**Authors:** Anna D. T. Barthel, Claudia Buengeler

**Affiliations:** Department of Human Resource Management and Organization, Faculty of Business, Economics and Social Sciences, Kiel University, Kiel, Germany

**Keywords:** servant leadership, directive leadership, leader promotability, leader effectiveness, leader liking, leader gender, gender stereotypes, expectancy violation theory

## Abstract

Are leaders more promotable when they show servant or directive leadership – and does this hold for women and men alike? Servant leaders are likely seen as more effective, likable, and thus promotable but less prototypical than directive leaders. We argue that differing degrees of communion (i.e., warmth, morality) and agency (i.e., competence, dominance) underlie the relationship of servant and directive leadership with leaders’ promotability. Based on expectancy-violation theory, we assume that men benefit more from servant leadership and women benefit more from directive leadership. Servant leadership aligns more with communion and stereotypes about women. In contrast, directive leadership aligns more with agency and stereotypes about men. These differences may result in gender-biased evaluations threatening fairness in leadership promotions. In a pre-study, servant leadership was more expected of women leaders than of men leaders. However, directive leadership was equally expected of women leaders and men leaders. An experimental vignette study (*N* = 454) revealed that servant leaders were seen as more effective, likable, and promotable than directive leaders, regardless of gender. Perceived leader warmth, morality, and competence were positively, and dominance was negatively, related to leader effectiveness and leader liking, which were positively related to leader promotability. We also investigated whether raters’ gender role beliefs influenced the evaluations, which they did not (as reported in the Supplementary material). Concluding, women and men profit equally from exhibiting servant compared to directive leadership.

## Introduction

There is ample evidence on what leadership behaviors benefit organizations and followers (e.g., Judge et al., 2004; Hoch et al., 2018) – but do these behaviors also support a leader’s career? Leaders may implement certain leadership behaviors more when these also benefit their promotion. Promotions are often based on evaluating a leader’s effectiveness and liking (e.g., Shaughnessy et al., 2011; Hentschel et al., 2018), and leadership behavior is central to this (e.g., Rojahn and Willemsen, 1994; DeRue et al., 2011; Hentschel et al., 2018). Research demonstrates that a follower-oriented leadership behavior, like servant leadership, positively relates to perceived leader effectiveness *and* liking (i.e., high LMX; Hoch et al., 2018; Zhang et al., 2021). *Servant leadership* captures empowering followers and putting their needs first (Eva et al., 2019). In contrast, a more task-oriented behavior like directive leadership primarily focuses on performance-related outcomes by giving orders and monitoring followers (House, 1971; Pearce and Sims, 2002). We argue that servant leaders will be seen as more promotable because they consider the needs of followers rather than only telling them what to do. Because of these behaviors, they will be seen as more effective in leadership and more likable than directive leaders. However, as directive leaders likely match the typical image of a leader (Northouse, 2016; Offermann and Coats, 2018) more than servant leaders, we expect that they will be seen as more prototypical.

Leader gender might bias the evaluation of servant versus directive leaders’ promotability. We expect that both leadership behaviors are contrary in whether they confirm or violate gender stereotypes. Gender stereotypes depict women as more *communal* (e.g., sensitive, nurturing; Eagly et al., 2020). Servant leadership comprises mainly communal, “feminine” behaviors like caring for followers that are more expected of women (Hogue, 2016; Eva et al., 2019). Men are stereotyped as more *agentic* (e.g., assertive, having leadership ability; Prentice and Carranza, 2002; Eagly et al., 2020). Directive leadership captures primarily agentic, “masculine” behaviors like giving orders (Eagly and Johnson, 1990; Pearce and Sims, 2002). Thus, women who show directive leadership and men who show servant leadership are likely perceived to violate gender-role-specific expectations.

Violating expectations either results in an evaluative penalty or a bonus (Jussim et al., 1987). A penalty occurs when an unexpected and negative behavior is shown. When women show agentic behavior that contradicts communion expectations, they are penalized as less likable and promotable than men because such behavior is deemed undesirable for women (*role congruity theory*, Eagly and Karau, 2002; e.g., Rudman et al., 2012; Ma et al., 2022). Similarly, when men show communal behavior that contradicts agency expectations, they are penalized as weak and less likable (Moss-Racusin et al., 2010; Hernandez Bark et al., 2022). A bonus occurs when an unexpected but positive behavior is shown (*expectancy-violation theory*, Jussim et al., 1987; Prentice and Carranza, 2004). Servant and directive leadership are positive behaviors because they benefit followers and organizations (Judge et al., 2004; Hoch et al., 2018). We propose that directive women leaders and servant men leaders exceed typical expectations positively. They are seen as combining communion with agency, or vice versa, resulting in a more favorable evaluation (Prentice and Carranza, 2004). Thus, we expect that directive women leaders receive an *agency bonus* compared to directive men leaders, while servant men leaders receive a *communion bonus* compared to servant women leaders. The bonus should be evident in higher ratings of leader effectiveness, liking, and promotability. Yet, due to the perceived incongruence of their leadership behavior with their gender role, directive women leaders and servant men leaders are likely deemed as less typical leaders. Thus, we expect them to receive lower ratings of leader prototypicality than stereotype-conforming leaders.

**Figure 1:**
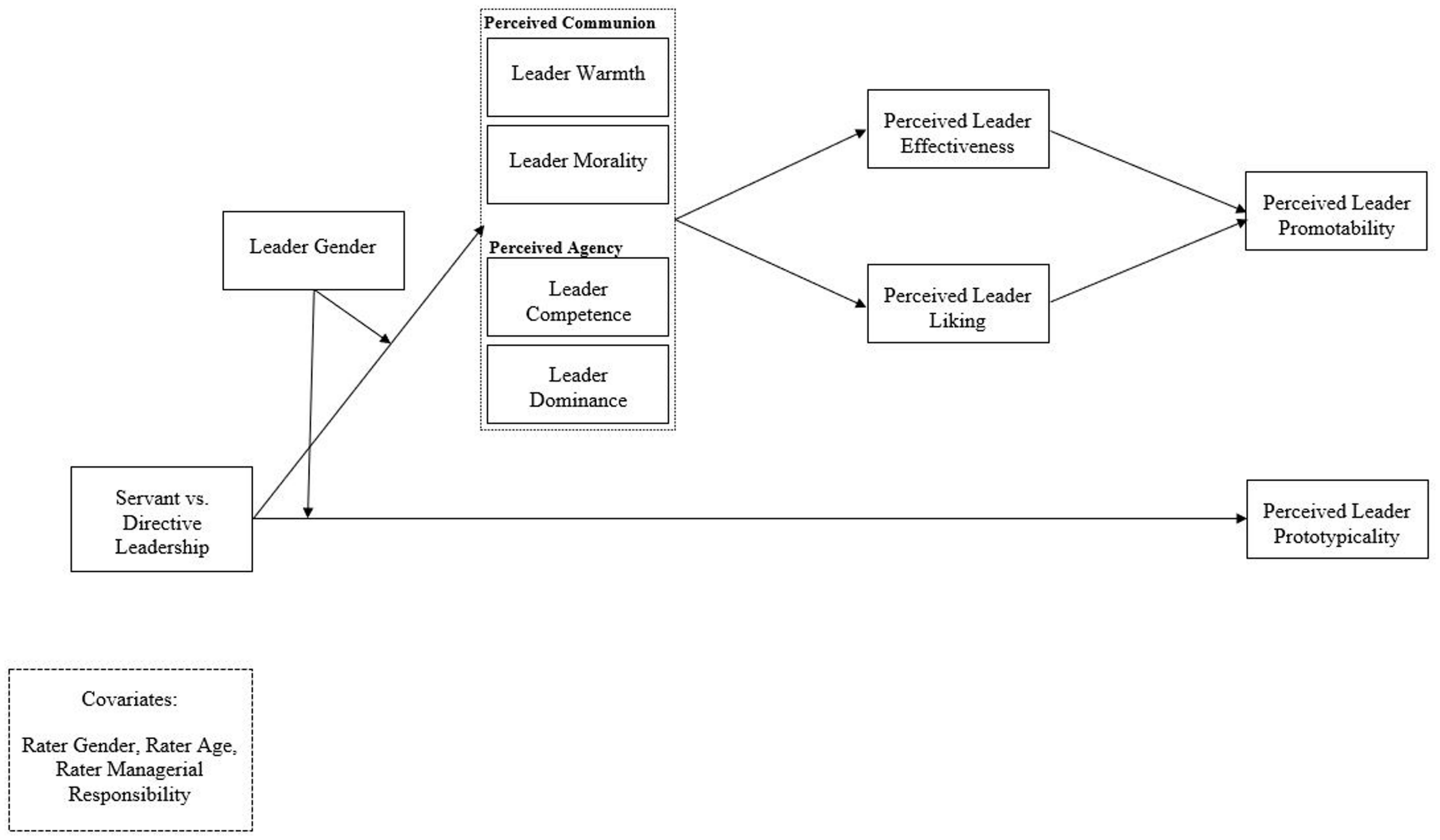
Hypothesized overall research model.

In conclusion, our first aim is to examine how servant and directive leadership relate to a leader’s promotability due to higher perceived leader effectiveness and liking. Differences in perceived leader communion and agency may drive these relationships. Thus, we examine whether communion and agency are the underlying mechanisms of these evaluations. We follow recent evidence (Hentschel et al., 2019; Ma et al., 2022) as we examine the facets of communion (i.e., warmth and morality) and agency (i.e., competence and dominance; Abele et al., 2016; Rosette et al., 2016) rather than less fine-grained overarching factors. Our second aim is to examine leader gender as a contingency factor, as research suggests that the evaluation of communal and agentic behavior varies according to leader gender (Biernat, 2012; Hentschel et al., 2018). We implement an experimental vignette study in which we manipulate leadership behavior and leader gender using written scenarios. Figure 1 summarizes the hypothesized overall research model.

Our research makes important contributions. First, we add to research on the outcomes of servant as compared to directive leadership by examining their relationships with leader promotability (Judge et al., 2004; Hoch et al., 2018; Eva et al., 2019). There is clear evidence on the benefits of servant leadership for individuals, teams, and organizations. Yet, it remains unclear whether servant leadership helps leaders to advance their careers. We contribute knowledge on how much leaders themselves profit from servant leadership compared to directive leadership, a more typical leadership behavior. In this regard, we shed light on whether servant leadership serves not only organizations and followers but also leaders.

Second, we clarify the mechanisms underlying servant and directive leadership evaluations. We examine whether perceptions of leaders’ communion and agency explain the relationship between leadership behavior and perceived leader effectiveness, liking, and promotability. Hereby, we expand knowledge on how leaders can be perceived as effective and likable to receive promotion (Rojahn and Willemsen, 1994; Gartzia and Van Knippenberg, 2016; Hentschel et al., 2018). We add to existing research and evidence on the benefits of examining the facets of communion and agency (Hentschel et al., 2019; Ma et al., 2022).

Third, we contribute to the literature on gender-biased leadership evaluations.^1^ We integrate expectancy-violation theory (Jussim et al., 1987; Prentice and Carranza, 2004) with role congruity theory (Eagly and Karau, 2002). Doing so, we theorize and provide evidence on whether and why women and men are rewarded for gender role-incongruent leadership behavior. As we examine evaluative consequences of servant leadership for women versus men, we answer calls for research on gender and servant leadership (Eva et al., 2019) and on male communion bonus in leadership (Hentschel et al., 2018). It is important to investigate whether men receive better evaluations than women for the same leadership behavior or vice versa because such bias threatens gender equity in leadership promotions and positions.

### Leadership behavior and leader evaluations

Leadership behaviors are behavioral patterns by which leaders seek to influence their followers (Yukl, 1989). Decades ago, McGregor (1960) proposed that leaders differ in their understanding of followers and how they must be led to produce the best results. Leaders could implement a follower-oriented approach by supporting the needs of followers or a task-oriented approach by directing and monitoring followers. While servant leadership is more follower-oriented and thus a communal leadership behavior (Hogue, 2016), directive leadership is more task-oriented and thus an agentic leadership behavior (Eagly and Johnson, 1990; Pearce and Sims, 2002).

#### Servant leadership

By putting followers first and focusing on their growth, servant leaders empower them to develop their best potential (Eva et al., 2019). In addition, servant leaders encourage followers to dedicate themselves beyond their self-interest to the wider community and organization (Eva et al., 2019). Servant leadership positively relates to follower’s job satisfaction, leader effectiveness, and leader liking (i.e., high LMX; Hoch et al., 2018; Zhang et al., 2021).

#### Directive leadership

Directive leadership involves setting goals, directing, and monitoring followers (House, 1996; Pearce and Sims, 2002). Directive forms of leading are positively related to follower job satisfaction, satisfaction with the leader, motivation, and leader effectiveness (House, 1971; Judge et al., 2004). However, directive leadership does not promote followers’ liking of their leader (Peterson, 1997).

#### Leadership behavior and promotability

Leader promotability is an important parameter for evaluating how much leaders themselves benefit from showing certain leadership behaviors. Leader promotability captures perceptions of a leader’s capability to ascend the organizational ladder (Ma et al., 2022). Communal behaviors more positively predict leader effectiveness than agentic behaviors (Judge et al., 2004). Because servant leaders support followers’ needs, they are likely seen as more effective and as more likable than directive leaders. Being seen as effective and likable positively relates to promotability (e.g., Shaughnessy et al., 2011; Hentschel et al., 2018). Thus, we argue that servant leadership is related to higher perceptions of leader effectiveness, liking, and promotability than directive leadership.

#### Leadership behavior and leader prototypicality

Leader prototypicality reflects how much a certain leadership behavior aligns with the typical image of a leader.^2^ Stereotypes toward leaders, so-called *leader prototypes* (implicit leadership theories; Lord et al., 1984; Offermann and Coats, 2018), reflect people’s shared beliefs about the characteristics of leaders and leadership behavior. Raters assess leaders’ prototypicality by comparing them with these leader prototypes (*leadership categorization theory*, Lord et al., 1984; Lord and Maher, 1991). Apart from communal characteristics like sensitivity or dedication, agentic characteristics like strength or tyranny are overrepresented in leader prototypes and still deemed more typical of a leader (Koenig et al., 2011; Offermann and Coats, 2018). Thus, we argue that servant leadership is perceived as less prototypical than directive leadership.

*Hypothesis* 1: Servant leadership compared to directive leadership relates more positively to perceived leader effectiveness (H1a), liking (H1b), and promotability (H1c) but less positively to perceived leader prototypicality (H1d).

### Leadership behavior, leader evaluations, and leader gender

#### Leadership behavior and leader gender

Leadership behaviors are perceived as more or less stereotypically “feminine” or “masculine” (see Kark et al., 2023) and thus as rather congruent or incongruent with gender stereotypes (Eagly and Karau, 2002). Servant leadership is congruent with the “feminine” gender role (Hogue, 2016; Lemoine and Blum, 2021), whereas directive leadership is congruent with the “masculine” gender role and the leader role (Eagly and Johnson, 1990; Eagly and Karau, 2002). Research suggests that women leaders are expected to enact more servant leadership, while men leaders are expected to show more agentic leadership behavior (Hogue, 2016), such as directive leadership. But how are women and men leaders evaluated when showing gender role (in)congruent leadership behavior?

Results on the evaluation of gender role (in)congruent behavior so far were inconsistent. Some research hints at a penalty evident in lower perceived liking and hireability ratings for agentic women than agentic men (e.g., Rudman, 1998; Rudman et al., 2012). Yet, recent research suggests a promotability bonus for agentic women (Ma et al., 2022). For communal men, some research suggests that these men were seen as less likable but not as less competent or hirable than communal women (Moss-Racusin et al., 2010; Hernandez Bark et al., 2022). Other research suggests a bonus for communal men leaders compared to communal women leaders in the form of higher perceived leader effectiveness and promotability (Hentschel et al., 2018).

#### Leadership behavior, leader gender, and expectancy-violation theory

We argue that servant men leaders and directive women leaders receive an evaluative bonus compared to stereotype-conforming leaders (servant women leaders and directive men leaders). To explain whether a bonus or penalty occurs, expectancy-violation theory (Jussim et al., 1987; Prentice and Carranza, 2004) distinguishes whether a *descriptive*, *prescriptive,* or *proscriptive* gender stereotype is violated (Prentice and Carranza, 2004). *Descriptive* gender stereotypes reflect how women/men typically are. *Prescriptive* gender stereotypes capture how women/men ideally should be. Finally, *proscriptive* gender stereotypes reflect how women/men ought not to be (Burgess and Borgida, 1999; Heilman, 2012; Rudman et al., 2012). A penalty occurs for violating prescriptive or proscriptive gender stereotypes, evident in lower social attractiveness and popularity (Eagly and Karau, 2002; Prentice and Carranza, 2004; see Rudman and Glick, 2001). A penalty also occurs for violating a descriptive gender stereotype by exhibiting a *negative* attribute deemed undesirable in society (Jussim et al., 1987; Prentice and Carranza, 2004). Yet, a bonus occurs when one violates descriptive gender stereotypes and thus raters’ expectations by exhibiting a *positive* attribute that is generally seen as desirable (Jussim et al., 1987; Bettencourt et al., 1997). Servant men leaders and directive women leaders violate the expectation that women are typically not agentic and that men are typically not communal. These violations likely result in a bonus, as servant and directive leadership are positive behaviors. Servant men leaders might be perceived as agentic (because of gender stereotypes) but also as communal (due to their leadership behavior). Directive women leaders might be seen as agentic (due to their leadership behavior) and as communal (because of gender stereotypes).

#### Leadership behavior, leader gender, leader evaluations, leader promotability, and prototypicality

We propose that servant men leaders and directive women leaders score higher on perceived leader effectiveness, liking, and promotability but lower on leader prototypicality than servant women leaders and directive men leaders. Violating descriptive stereotypes has a more extreme impact on evaluations than confirming stereotypes (Jussim et al., 1987). Thus, servant men leaders and directive women leaders are likely seen as more effective and likable than their stereotype-conforming counterparts. Since leader effectiveness and liking are related to promotability (Shaughnessy et al., 2011; Hentschel et al., 2018), we expect that this evaluative bonus is also evident in leaders’ promotability. Yet, due to the perceived incongruence between servant leadership behavior and men’s agentic gender roles, we expect servant men leaders to score lower on perceived leader prototypicality than servant women leaders. Due to the perceived incongruence between directive leadership behavior and women’s communal gender roles, we expect directive women leaders to score lower on perceived leader prototypicality than directive men leaders.

*Hypothesis* 2: For men (women) leaders as compared to women (men) leaders, servant (directive) leadership relates more positively to perceived leader effectiveness (H2a), liking (H2b), and promotability (H2c) but less positively to perceived leader prototypicality (H2d).

### The mediating role of perceived communion and agency

#### Servant and directive leadership, communion, agency, and leader promotability

We propose that communion and agency underlie the relationship of servant and directive leadership behavior with perceived leader effectiveness and liking, which, in turn, predict leader promotability. Communion and agency are composed of facets. Distinguishing these facets offers a more differentiated view because the facets differ in their social desirability and whether they are prescribed or proscribed for women and men (Rudman et al., 2012; Hentschel et al., 2019; Ma et al., 2022). As a result, the facets differ according to whether a positive or negative violation occurs.

#### Communion and agency

Communion contains warmth and morality (Abele et al., 2008, 2016). *Warmth* is the ability to connect and cooperate with other people, while *morality* captures a person’s perceived trustworthiness and correctness (Brambilla et al., 2011). Communion is linked to be seen as effective, and likable, and to leader promotability (Wojciszke et al., 2009; Hentschel et al., 2018). Agency generally comprises competence (Abele et al., 2016, 2021) and dominance (Rudman and Glick, 2001; Rosette et al., 2016). *Competence* refers to a person’s task-based talents and skills (Abele et al., 2016) and relates to a person’s perceived leader effectiveness, liking, and promotability (Singh and Tor, 2008; Dulebohn et al., 2017; Hu et al., 2022; Ma et al., 2022). *Dominance* is a person’s tendency to control and exercise influence and authority over others (Rosette et al., 2016). Dominance is part of destructive leadership (Padilla et al., 2007) and, unsurprisingly, holds a null or negative relationship with perceived leader liking (Cheng et al., 2013), and a negative relationship with leader promotability (Ma et al., 2022).

#### Leadership behavior, communion, and gender-biased leadership evaluations

We argue that leader gender influences the relationship of servant and directive leadership with communion in terms of perceived warmth and morality. We expect that servant leadership positively predicts perceptions of leader warmth and morality as servant leadership is a communal leadership behavior. Warmth and morality include behaviors prescribed for women but not proscribed for men (Prentice and Carranza, 2002; Abele et al., 2008, 2021). Thus, following the assumptions of expectancy-violation theory (Prentice and Carranza, 2004) and role congruity theory (Eagly and Karau, 2002), we argue that women displaying servant leadership evade a penalty as servant leadership aligns with communion. We expect that servant men leaders receive a bonus as they positively violate expectations that they are low on communion. Interestingly, women and men are evaluated according to stereotypes for their gender group (*shifting standards theory*; Biernat, 2012). Men showing warmth and morality are likely perceived as especially warm and moral for men, while women doing the same are perceived as averagely warm and moral for women. Thus, we expect that the positive relationship of servant vs. directive leadership with warmth and morality is stronger for men leaders compared to women leaders. Being seen as warm and moral positively relates to leader effectiveness, liking, and, consequently, promotability (Wojciszke et al., 2009; Shaughnessy et al., 2011; Hentschel et al., 2018). Thus, we propose:

*Hypothesis* 3: Servant (compared to directive) leadership positively relates to perceptions of leader warmth (H3a) and morality (H3b), which, in turn, positively relate to perceived leader effectiveness and liking, and, ultimately, to promotability. These mediation effects are stronger for men leaders compared to women leaders.

#### Leadership behavior, agency, and gender-biased leadership evaluations

We argue that leader gender influences the relationship of servant and directive leadership with perceived competence and dominance. We expect that servant leadership positively relates to perceived leader competence and negatively to dominance. Competence and dominance differ in their gendered prescription and proscription. Omitting these differences in previous research and that competence is socially desirable while dominance is undesirable might be one reason for inconclusive findings regarding women’s agency bonus and penalty (Ma et al., 2022). Competence is prescribed for men but neither prescribed nor proscribed for women (Rudman et al., 2012). Thus, women leaders receive a bonus for displaying competence (Prentice and Carranza, 2004; e.g., Ma et al., 2022). Dominance is prescribed for men given their higher status in society but proscribed for women given their lower status in society (*status incongruity hypothesis*, Rudman et al., 2012). Thus, dominance is even more negatively linked to perceived promotability for women leaders than for men leaders (Ma et al., 2022). Due to shifting gender standards for competence (Biernat, 2012), women displaying competence are likely perceived as especially competent for women, while men doing the same are perceived as averagely competent for men. Thus, we propose that the positive relationship between servant vs. directive leadership behavior and competence is stronger for women leaders than for men leaders. As men in general are seen as more dominant than women due to gender stereotypes (i.e., agentic; Eagly et al., 2020), servant men leaders are likely perceived as more dominant than servant women leaders. Thus, we expect that the negative relationship between servant vs. directive leadership behavior and dominance is stronger for women leaders than for men leaders. Being seen as competent positively, and as dominant negatively, relates to leader effectiveness, liking, and, consequently, promotability (Singh and Tor, 2008; Shaughnessy et al., 2011; Cheng et al., 2013; Dulebohn et al., 2017; Hentschel et al., 2018; Hu et al., 2022; Ma et al., 2022). Thus, we propose:

*Hypothesis* 4: Servant (compared to directive) leadership positively relates to perceptions of leader competence (H4a) and negatively relates to perceptions of leader dominance (H4b). In turn, competence positively and dominance negatively relate to perceived leader effectiveness and liking, and, ultimately, to promotability. These mediation effects are stronger for women leaders compared to men leaders.

## Overview of studies

Before testing our hypotheses, we conducted two pre-studies. Pre-study 1 concerns gender-biased leadership expectations. Pre-study 2 validates the visual stimulus material of two silhouettes used in the main study.

Additional analyses and results regarding our hypothesis about the moderating role of raters’ gender role beliefs can be found in the Supplementary material (section #3.2.2). Raters may differ whether they evaluate a gender stereotype violation as positive or negative depending on their gender role beliefs (role congruity theory, Eagly and Karau, 2002). We assessed raters’ egalitarian gender role beliefs via Larsen and Long’s (1988) 20-item comprising *Attitudes toward Sex Roles Scale*. The results did not support our hypothesis about raters with traditional beliefs giving more unfavorable and with egalitarian beliefs giving more favorable evaluations for stereotype-violating leaders compared to stereotype-conforming leaders. Due to the high skewness of our data toward egalitarian gender role beliefs, the analyses and results must be interpreted with caution and were moved to the Supplementary material during the revision process. Thus, we can neither support nor reject the suggestion that raters’ gender role beliefs may evoke gender bias in the evaluation of servant or directive leaders. In the Supplementary material (section #3.2.2), we further elaborate on the potential demand effects that might have occurred for the scale of gender role beliefs.

### Pre-study 1

#### Expectations of leader gender and leadership behaviors

We investigated whether women are expected to exhibit more servant leadership than men and whether men are expected to show more directive leadership than women. Specifically, we investigated descriptive, *typical* leadership expectations representing leadership behaviors that women and men are expected to show typically. We also investigated prescriptive, *ideal* leadership expectations representing leadership behaviors women and men should ideally show.

#### Method

We conducted a 2 (leader gender: woman, man) × 2 (expectation: typical, ideal) experiment with leader gender varying within-subject and expectations varying between-subject. We recruited an online sample in Germany and randomly assigned participants to one of two conditions, *typical* (*N* = 44, 70.5% female, 2 participants did not indicate their gender, *M*_age_ = 28.95 years, *SD*_age_ = 8.97) or *ideal* (*N* = 48, 77.1% female, *M*_age_ = 28.65 years, *SD*_age_ = 9.82) leadership expectations. In each condition, we randomized whether participants had to first answer for women leaders or men leaders, with a distractor task in-between (see Supplementary material, section #1.2). The instruction for the typical/ideal condition was: “The following refers to your expectations regarding typical/ideal behavior. Please imagine having a woman/man as your formal supervisor.” We chose this instruction as imagining a woman/man as formal supervisor corresponds to the scenario manipulation we used in the main study. The typical condition represented descriptive leadership stereotypes (“What kind of leadership behavior does a woman/man typically exhibit?”), while the ideal condition represented prescriptive leadership stereotypes (“What kind of leadership behavior does a woman/man ideally exhibit?”). *Servant leadership* was operationalized by the seven items of the SL-7 (Liden et al., 2015; Ruthus, 2019), with one item being adapted to “I can seek help from her/him if I have a personal problem.” *Directive leadership* was measured by five items taken and adapted from Northouse (2016; e.g., “She/He lets me know what is expected of me”).^3^ Participants indicated on a 7-point Likert scale from (1) *do not agree at all* to (7) *totally agree* on how much they agreed with the presented leadership items.

#### Do people believe women to (typically and ideally) show more servant leadership? Results

**Figure 2:**
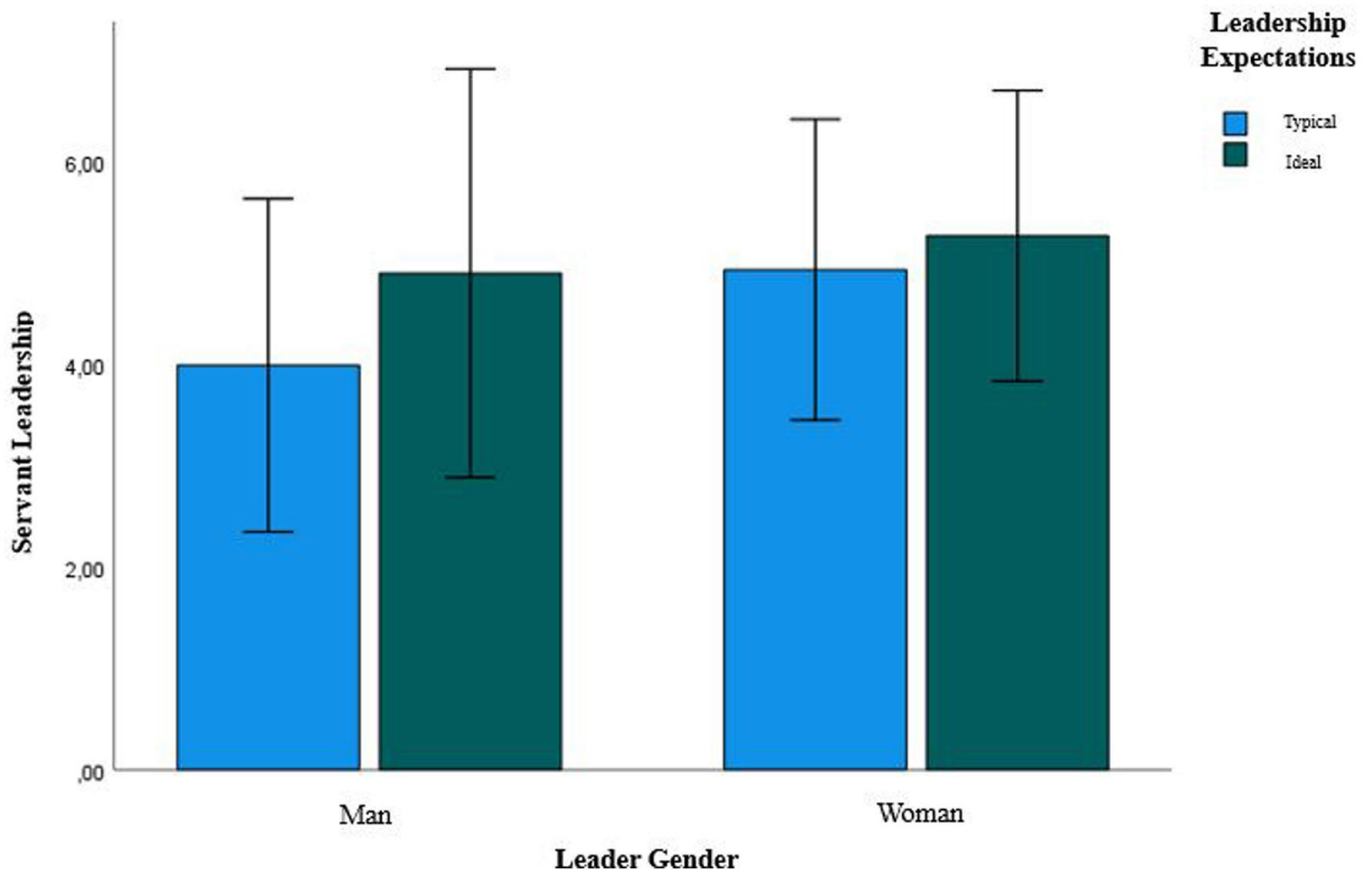
Bar plot showing how much servant leadership is typically and ideally expected of women leaders and men leaders.

Concerning *typical* leadership expectations, the paired *t*-test indicated that women were expected to typically show more servant leadership (*M* = 4.93, *SD* = 0.74) than men (*M* = 3.98, *SD* = 0.82, *t*(43) = 8.12, *p* < 0.001). Concerning *ideal* leadership expectations,^4^ women should ideally show more servant leadership (*M* = 5.26, *SD* = 0.72) than men (*M* = 4.89, *SD* = 1.01, *t*(47) = 3.53, *p* < 0.01; see Figure 2).

#### Do people believe men to (typically and ideally) show more directive leadership? Results

**Figure 3:**
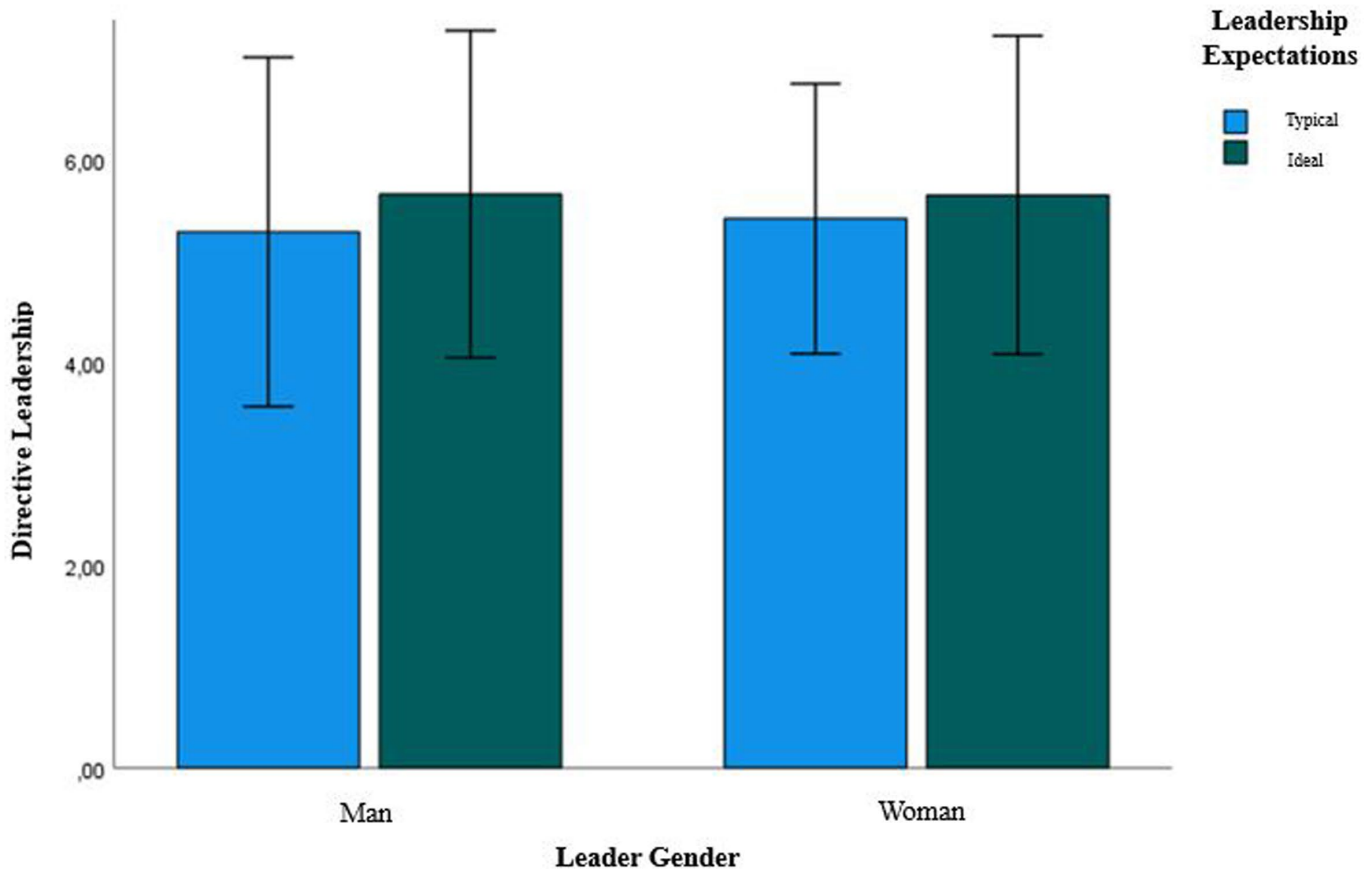
Bar plot showing how much directive leadership is typically and ideally expected of women leaders and men leaders.

Concerning *typical* leadership expectations, women (*M* = 5.41, *SD* = 0.66) and men (*M* = 5.28, *SD* = 0.86, *t*(43) = 1.29, *p* = 0.20) were equally expected to typically show directive leadership. We also found no differences in *ideal* leadership expectations as men (*M* = 5.65, *SD* = 0.81) and women (*M* = 5.64, *SD* = 0.78, *t*(47) = 0.09, *p* = 0.93; see Figure 3) were equally expected to ideally show directive leadership.

### Pre-study 2

#### Validation of silhouettes

To increase gender salience regarding our leadership scenario in the main study, we validated female and male visual stimuli for leader gender. One more realistic option was a female and a male face (following Buengeler et al., 2016; generated from several pictures taken from DeBruine and Jones (2017) using the tool *WebMorph* (DeBruine, 2018), see Supplementary material, section #2.2). The other more abstract option was a female and a male silhouette (adapted from Hernandez Bark et al., 2022). We adapted the female silhouette by inserting it in a blank background so that it was presented on a blank background like the male silhouette.

#### Method

We recruited an online sample in Germany (*N* = 42, 52.4% female, 38.1% male, 4.8% diverse, 4.8% did not specify their gender; *M*_age_ = 38.27 years (*SD* = 14.62), 1 participant did not indicate the own age). We presented participants with a female and a male person via a face and a silhouette. Participants saw the female and male face as well as the female and male silhouette. We randomly assigned whether the faces or silhouettes were presented first. Within the faces and silhouettes condition we randomized which gender was displayed first. A distractor task was inserted between the faces and silhouettes (see Supplementary material, section #2.2). Regarding the presented stimuli, participants indicated the person’s perceived attractiveness, intelligence, liking, dominance, competence, warmth, and morality, as well as how friendly they would treat the person and how much they would be interested in getting to know the person on a 5-point Likert scale. Additionally, they indicated the perceived age and perceived gender of the presented stimuli. The questions were created by the authors, were presented in a randomized order, and are listed in the Supplementary material (section #2.2).

#### Results

The female and the male face differed substantially (see results in the Supplementary material, section #2.3), so we focused on silhouettes for the stimulus material. The paired *t*-tests indicated no differences between the silhouettes except regarding their perceived gender and gender stereotypes (see Table 1). The female silhouette was perceived to be warmer and more moral than the male. The male silhouette was perceived to be more dominant.

**Table 1:** Means and standard deviations for the female silhouette and the male silhouette.

Measures	Condition				
	Female		Male		
	*M*	*SD*	*M*	*SD*	Paired *t*-test
Perceived attractiveness	3.45	0.63	3.40	0.73	*t*(41) = 0.42, *p* = 0.68
Perceived intelligence	3.62	0.54	3.67	0.65	*t*(41) = 0.63, *p* = 0.53
Perceived liking	3.17	0.49	3.00	0.63	*t*(41) = 1.64, *p* = 0.11
Interest in getting to know the person	3.12	0.74	2.81	0.92	*t*(41) = 1.87, *p* = 0.07
Treating the person in a friendly manner	3.69	0.47	3.64	0.49	*t*(41) = 0.81, *p* = 0.42
Perceived dominance	3.26	0.63	3.62	0.62	*t*(41) = 2.64*, *p* = 0.01
Perceived competence	3.69	0.64	3.60	0.73	*t*(41) = 0.94, *p* = 0.35
Perceived warmth	3.02	0.52	2.69	0.64	*t*(41) = 2.65*, *p* = 0.01
Perceived morality	3.21	0.68	2.98	0.72	*t*(41) = 2.23**, *p* = 0.03
Perceived gender	1.10	0.37	0.29	0.71	*t*(41) = 11.54**, *p* < 0.001
Perceived age	35.65^a^	5.24	36.86^a^	6.20	*t*(36) = 1.38, *p* = 0.18

For perceived gender, male is coded as 0, female as 1, and I do not know as 2. *N* = 42.

a *N* = 37, *p < 0.05; **p < 0.01.

## Method and materials

### Sample and design

To test our hypotheses, we conducted an experimental online study. The 2 × 2 design is based on two independent variables: leader’s leadership behavior (servant vs. directive leadership) and leader gender (woman vs. man). The total sample consisted of *N* = 454 full-time working employees (>29 h/week) after excluding participants who failed manipulation and quality checks during the survey (see procedure and manipulation, see manipulation checks).^5^ Participants needed to be German native speakers to ensure their susceptibility to the gendered language used in the study’s manipulation and questionnaires.^6^ Two hundred twenty seven participants were women (50.00%), and the mean age was 45.92 years (*SD* = 11.66). On average, participants worked 39.68 h per week (*SD* = 4.71). Participants’ highest education was vocational training (27.1%), intermediate school leaving certificate (19.4%), university of applied sciences degree (18.7%), advanced school leaving certificate (14.1%), university degree (14.1%), lower secondary education (5.1%), and a Ph.D. degree (1.5%). One hundred twenty one participants held a supervisory position themselves (26.7%). Most participants indicated that they have or had a formal supervisor at work (96.5%). Two hundred fifty five of the participants indicated that they thought about their (former) supervisor during the survey (56.2%), while 199 participants indicated that they did not think about any (former) supervisor (43.8%, 1 of these never had any supervisor). Most participants indicated that they could imagine the presented scenario very well, well, or moderately well (93.1%); only 31 participants found it difficult or very difficult (6.9%).^7^

### Procedure and manipulation

In the online survey, we informed participants that we were interested in their evaluation of a leadership scenario. Then, they were divided according to their gender and randomly assigned to one of four manipulation conditions, to ensure a nearly equal number of women and men across conditions. We took this measure to counterbalance participant gender. All participants then read the instruction: “The following text describes a situation in the workplace. Please read the text on the next page carefully. It is important that you put yourself in the scenario described. Please imagine that the person described is your formal supervisor in real life.”

Each condition included a written scenario in which the respective supervisor was either a woman or a man. The female or male silhouette accompanied scenarios to increase gender salience (see Pre-Study 2). In addition, we used the gendered nature of the German language. The female or male version of “supervisor” highlighted the respective gender and was accompanied by the gendered possessive pronoun “your” (*Ihre Vorgesetzte* or *Ihr Vorgesetzter)*. The translated version of the scenarios is depicted in Table 2. Each scenario started with “You work full time in an organization. In the picture, you see your formal supervisor.” The female or male silhouette was presented below, followed by the manipulation of the leadership behavior and leader gender.

**Table 2:** English version of written scenarios for the manipulation.

Leadership behavior	
Servant leadership	Directive leadership
*Your supervisor* makes your career development a priority. *Your supervisor* emphasizes the importance of giving back to the community and puts the interests of *her/his* subordinates above *her/his* own. If you have a personal problem, you can seek help from *her/him*. *Your supervisor* recognizes when something work-related is going wrong. *Your supervisor* gives you the freedom to handle difficult situations in the way that you feel is best. *S/he* would *not* compromise ethical principles in order to achieve success.	*Your supervisor* lets you know what is expected of you. *Your supervisor* tells you what needs to be done and how it needs to be done. *Your supervisor* asks you to follow standard rules and regulations. *S/he* makes it clear to each of *her/his* subordinates what his or her role is in the group. *Your supervisor* explains the level of performance *s/he* expects from you. *S/he* would never give vague explanations about what is expected of you on the job.

The words that were gendered in the original German manipulation are highlighted in italics. The written manipulation of the leadership behavior was derived from the respective short scales of servant leadership (SL-7, Liden et al., 2015) and the five items for measuring directive leadership (Northouse, 2016). We created another item for the scenario about directive leadership to ensure an equal length of both leadership scenarios (e.g., “She makes it clear to each of her subordinates what his or her role is in the group.”). The scenarios were written in German and equal in length, cues for leader gender, and cues addressing the reader. See Supplementary material (section #3.1) for the German scenarios.

### Manipulation checks

We employed instructional manipulation checks regarding the characteristics of the person described in the scenario to ensure that participants understood the manipulation correctly (Oppenheimer et al., 2009). We asked for the gender (woman, man, no gender mentioned, I do not know), the organizational position (own supervisor, colleague, CEO of company), and the general leadership behavior (follower-focused, task-focused, I do not know) of the person depicted in the scenario (see Supplementary material, section #3.1). Participants who failed to respond correctly could not continue with the survey.^8^ In addition, we were interested in how much participants perceived the described supervisor to exhibit servant and/or directive leadership. Participants assessed the perceived leadership behavior by replying to four items for each behavior on a 7-point Likert scale (1 = *do not agree at all* to 7 = *totally agree*). From the SL-7 we selected four items with the highest factor loadings (Liden et al., 2015; items 2, 3, 5, 6). From the directive leadership items (Northouse, 2016), we took all but the reversed item because of its’ low item-total correlation in Pre-Study 1. A Welch-test showed differing servant leadership perceptions between the servant and directive leadership condition (95% CI [−3.44, −3.04], *t*(439.05) = −32.23, *p* < 0.001). We also found differing directive leadership perceptions between the servant and directive leadership condition (95% CI [1.27, 1.66], *t*(451.38) = 14.94, *p* < 0.001; for means per condition, see Table 3).^9^

**Table 3:** Means and standard deviations of perceived servant leadership and directive leadership per condition.

	Servant leadership						Directive leadership					
	Woman leader			Man leader			Woman leader			Man leader		
	*N*	*M*	*SE*	*N*	*M*	*SE*	*N*	*M*	*SE*	*N*	*M*	*SE*
Perceived servant leadership	126	5.76	0.94	98	6.02	0.97	125	2.59	1.18	105	2.69	1.16
Perceived directive leadership		4.65	0.98		4.66	1.14		6.11	1.15		6.14	0.90

Ratings were given on a 7-point scale with higher scores indicating high levels of the respective variable.

### Measures

The survey was conducted in German. We used German translations or used a back-translation procedure (Brislin, 1970) to translate the scales into German. We adapted the scales using the gendered version of “supervisor” to increase gender salience. For each scale, items were presented in randomized order. If not stated differently, participants responded on a 7-point Likert scale (1 = *do not agree at all* to 7 = *totally agree*).

#### Perceived leader effectiveness

We measured perceived leader effectiveness (α = 0.96) with two items adapted from Gündemir et al. (2019, e.g., “This supervisor is a good leader.”) and four items adapted from Rink et al. (2013, e.g., “This supervisor can instigate change.”).

#### Perceived leader liking

*Liking* was operationalized using the nine-item scale of Montoya and Horton (2004), who adapted Byrne and Wong’s (1962) Interpersonal Judgment Scale. We adapted the wording of the items to match the written scenario and to increase gender salience in German (e.g., “I would like to meet this supervisor.”). We adapted the scale’s general response range to a 7-point Likert scale (ranging for most items from 1 = *do not agree at all* to 7 = *entirely agree*; α = 0.97).

#### Perceived leader promotability

*Promotability* was assessed by three items (α = 0.90) adapted from Hentschel et al. (2018, e.g., “This supervisor should be recommended for a promotion.”).

#### Perceived leader prototypicality

*Leader prototypicality* was measured by four items (α = 0.94). Two items were adapted from Gündemir et al. (2019, e.g., “To what degree does this supervisor fit the image of a typical leader?”; 1 = *not very typical* to 7 = *very typical*). To include a behavioral component, we further added the items “To what degree does this supervisor act like a typical leader?” and “To what degree does this supervisor behave like a typical leader?” (1 = *not at all* to 7 = *entirely*).

#### The facets of perceived leader warmth, morality, competence, and dominance

We operationalized the facets of perceived leader communion, *warmth* (α = 0.96) and *morality* (α = 0.92), as well as perceived leader agency, *competence* (α = 0.92) and, for the sake of completeness of this measurement tool, we also assessed for exploratory analyses another facet of agency, *assertiveness* (α = 0.82, see Supplementary material, section #3.2.1), by Abele et al.’s (2016) validated German scale. The scale comprised five items per facet. Responses to the question “The supervisor in the scenario seems to be …” were given on a bipolar five-point scale, with 5 indicating high levels of the respective facet.

We assessed *dominance* via the five items of the stereotype category dominance (Rosette et al., 2016, e.g., “bossy”). Participants responded to “The supervisor in the scenario seems to be …” on a 5-point Likert scale (1 = *do not agree at all* to 5 = *entirely agree*; α = 0.91; the item “demanding” was excluded as the corrected item-total correlation was below 0.30 and Cronbach’s alpha was better when the item was deleted; Field, 2018).

#### Rater characteristics as control variables

Rater characteristics may influence the stereotypical perception of women and men. For *rater gender*, research found differences in the prevalence of gender stereotypes for female and male raters as well as men perceiving men in general to possess more leadership competence compared to women in general (Hentschel et al., 2019). Thus, male raters may perceive women leaders to score lower on competence compared to female raters. Similarly, *rater age* may influence the reaction toward women leaders and men leaders. Social role theory proposes that social roles change over time (Eagly and Wood, 2012) and research supports the change of gender stereotypes over time (Eagly et al., 2020). We controlled for rater age as older people potentially may hold more traditional gender role beliefs than younger people and may respond more negatively to a woman as a leader. Finally, we asked whether raters have *managerial responsibility* themselves as this might influence their leadership evaluation. Raters in supervisory roles may prefer their ingroup (leaders) over the outgroup (followers) due to in-group bias (*social identity theory*; Tajfel and Turner, 1986). Thus, they may rate other leaders more favorably. The results of our analyses did not differ when rerunning the analyses without these control variables (Becker et al., 2016; Bernerth and Aguinis, 2016).

## Results

Table 4 depicts the correlations of all dependent variables, mediators, and the covariates. For the full correlation table including all demographics, please see the Supplementary material (section #3.2). Table 5 depicts the means and standard deviations of the dependent variables and mediators for each condition.^10^

**Table 4:** Means, standard deviations, and correlations of the dependent variables, mediators, and covariates.

		*M*	*SD*	1	2	3	4	5	6	7	8	9	10
1	Leader effectiveness	4.96	1.51										
2	Leader promotability	4.85	1.62	0.86^**^									
3	Leader liking	4.46	1.64	0.84^**^	0.85^**^								
4	Leader prototypicality	4.19	1.44	0.14^**^	−0.01	−0.06							
5	Leader warmth	3.28	1.19	0.66^**^	0.68^**^	0.78^**^	−0.23^**^						
6	Leader morality	3.68	0.99	0.75^**^	0.74^**^	0.79^**^	−0.06	0.85^**^					
7	Leader competence	3.74	0.91	0.73^**^	0.68^**^	0.65^**^	0.18^**^	0.61^**^	0.80^**^				
8	Leader dominance	2.61	1.13	−0.70^**^	−0.74^**^	−0.81^**^	0.23^**^	−0.82^**^	−0.75^**^	−0.50^**^			
9	Rater gender	0.50	0.50	0.10^*^	0.10^*^	0.09^*^	0.00	0.04	0.08	0.09	−0.07		
10	Rater age	45.92	11.66	−0.10^*^	−0.12^*^	−0.08	−0.04	−0.08	−0.11^*^	−0.10^*^	0.06	−0.22^**^	
11	Rater’s managerial responsibility	0.27	0.44	−0.12^**^	−0.09	−0.04	−0.03	−0.04	−0.06	−0.12^**^	0.05	−0.11^*^	0.06

*N* = 454. Men are coded as 0, women are coded as 1. Age in years. Rater’s managerial responsibility, no coded as 0, yes coded as 1. Leader liking refers to rater’s perceived liking of the leader.

*p < 0.05; **p < 0.01.

**Table 5:** Means and standard deviations of the dependent variables and mediators per condition.

	Servant leadership				Directive leadership			
	Woman leader^a^		Man leader^b^		Woman leader^c^		Man leader^d^	
	*M*	*SE*	*M*	*SE*	*M*	*SE*	*M*	*SE*
Leader effectiveness	5.72	0.97	5.80	1.11	4.12	1.49	4.28	1.52
Leader liking	5.49	0.92	5.59	1.12	3.36	1.42	3.49	1.49
Leader promotability	5.79	1.07	5.78	1.23	3.84	1.54	4.03	1.42
Leader prototypicality	3.81	1.30	3.46	1.44	4.70	1.31	4.73	1.32
Leader warmth	4.09	0.84	4.24	0.74	2.39	0.86	2.48	0.83
Leader morality	4.19	0.83	4.32	0.73	3.12	0.85	3.15	0.84
Leader competence	4.01	0.80	4.06	0.83	3.41	0.89	3.49	0.94
Leader dominance	1.80	0.75	1.72	0.66	3.46	0.83	3.39	0.82

Perceived leader effectiveness, promotability, liking, and prototypicality were rated on a 7-point Likert scale, while perceived leader warmth, morality, competence, and dominance were rated on a 5-point Likert scale. In both cases, higher scores indicate high levels of the respective variable. Leader liking refers to the rater’s perceived liking of the leader. Means are adjusted for the covariates rater gender, rater age, and raters’ managerial responsibility.

a *N* = 126.

b *N* = 98.

c *N* = 125.

d *N* = 105.

### Test of gender-biased leader evaluation: effectiveness, liking, promotability, and prototypicality

To test H1 and H2,^11^ we computed univariate analyses of covariance (ANCOVAs) in SPSS 27 to examine the main effects of the leadership behavior (coded as 0 = directive, 1 = servant), leader gender (coded as 0 = man, 1 = woman), and their interaction across the single dependent variables. As covariates, we included rater gender, rater age, and rater managerial responsibility.

*H*1: Servant leadership compared to directive leadership relates more positively to perceived leader effectiveness (H1a), liking (H1b), and promotability (H1c) but less positively to perceived leader prototypicality (H1d).

Servant leaders received significantly higher ratings of perceived leader effectiveness (*F*(1, 447) = 166.53, *p* < 0.001, partial *η*^2^ = 0.27), liking (*F*(1, 447) = 319.92, *p* < 0.001, partial *η*^2^ = 0.42), and promotability (*F*(1, 447) = 220.28, *p* < 0.001, partial *η*^2^ = 0.33) than directive leaders. In addition, we computed pairwise comparisons due to heterogeneity of cell variances indicated by Levene’s test and used the robust method bootstrapping (1,000 resamples, Field, 2018). Pairwise comparisons revealed that servant leaders were perceived as more effective (*M_SL_* = 5.76, *M_DL_* = 4.21, 95% CI [1.32, 1.79], *p* < 0.01), more likable (*M*_SL_ = 5.54, *M*_DL_ = 3.43, 95% CI [1.90, 2.32], *p* < 0.01), and more promotable (*M*_SL_ = 5.78, *M*_DL_ = 3.94, 95% CI [1.58, 2.07], *p* < 0.01) than directive leaders.^12^ For perceived leader prototypicality, no further analyses were computed.^13^ H1a to H1c were supported as we found a main effect of leadership behavior on leader effectiveness, liking, and leader promotability.

*H*2: For men (women) leaders as compared to women (men) leaders, servant (directive) leadership relates more positively to perceived leader effectiveness (H2a), liking (H2b), and promotability (H2c) but less positively to perceived leader prototypicality (H2d).

We found no significant main effect of leader gender indicating women and men were not evaluated differently on perceived leader effectiveness (*F*(1, 447) = 1.91, *p* = 0.17, partial *η*^2^ = 0.00), liking (*F*(1, 447) = 1.44, *p* = 0.23, partial *η*^2^ = 0.00), and promotability (*F*(1, 447) = 1.11, *p* = 0.29, partial *η*^2^ = 0.00). We also found no interaction effect of leadership behavior and leader gender on perceived leader effectiveness (*F*(1, 447) = 0.06, *p* = 0.80, partial *η*^2^ = 0.00), liking (*F*(1, 447) = 0.01, *p* = 0.93, partial *η*^2^ = 0.00), and promotability (*F*(1, 447) = 0.40, *p* = 0.53, partial *η*^2^ = 0.00) indicating that women and men were not evaluated differently for exhibiting either servant or directive leadership. Thus, H2 was not supported.

### Test of moderated mediation: warmth, morality, competence, and dominance as mediators

*H*3: Servant (compared to directive) leadership positively relates to perceptions of leader warmth (H3a) and morality (H3b), which, in turn, positively relate to perceived leader effectiveness and liking, and, ultimately, to promotability. These mediation effects are stronger for men leaders compared to women leaders.

*H*4: Servant (compared to directive) leadership positively relates to perceptions of leader competence (H4a) and negatively relates to perceptions of leader dominance (H4b). In turn, competence positively and dominance negatively relate to perceived leader effectiveness and liking, and, ultimately, to promotability. These mediation effects are stronger for women leaders compared to men leaders.

To test H3 to H4, we used the PROCESS macro, version 4.2 (Hayes, 2018) in SPSS 27 to compute all moderated mediation analyses. We tested hypotheses using bootstrapping (5,000 resamples) with 95% bias-corrected confidence intervals of the hypothesized indirect effects. The same covariates were used. As independent variable, we included leadership behavior. The moderator variable was leader gender. To test the serial moderated mediation model of H3 through H4, we implemented model 83, with either perceived leader warmth, morality, competence, or dominance as first mediator and either perceived leader effectiveness or liking as second mediator, and perceived leader promotability as dependent variable. We z-standardized all continuous variables to account for the different scale ranges of the mediators.

For H3 to H4, we found support for the proposed positive indirect effect of leadership behavior on perceived leader promotability via perceived warmth (H3a), morality (H3b), competence (H4a) and for the proposed negative indirect effect via perceived dominance (H4b) for women and men leaders via perceived leader effectiveness and liking (see Table 6 for the indirect effects, see the Supplementary material for the PROCESS output of each serial moderated mediation, Supplementary material, section #3.2). Leadership behavior positively predicted perceived warmth (*B* = 1.49, *SE* = 0.09, 95% CI [1.31;1.66]), morality (*B* = 1.20, *SE* = 0.11, 95% CI [0.98; 1.41]), competence (*B* = 0.62, *SE* = 0.13, 95% CI [0.36; 0.89]), and negatively predicted perceived dominance (*B* = −1.47, *SE* = 0.09, 95% CI [−1.66; −1.29]). Warmth positively predicted effectiveness (*B* = 0.59, *SE* = 0.07, 95% CI [0.46; 0.72]) and liking (*B* = 0.65, *SE* = 0.06, 95% CI [0.53; 0.79]) which positively predicted leader promotability (effectiveness: *B* = 0.71, *SE* = 0.04, 95% CI [0.64; 0.79]; liking: *B* = 0.81, *SE* = 0.05, 95% CI [0.72; 0.90]). Morality positively predicted effectiveness (*B* = 0.66, *SE* = 0.06, 95% CI [0.54; 0.78]) and liking (*B* = 0.62, *SE* = 0.05, 95% CI [0.52; 0.73]) which positively predicted leader promotability (effectiveness: *B* = 0.66, *SE* = 0.04, 95% CI [0.58; 0.74]; liking: *B* = 0.71, *SE* = 0.05, 95% CI [0.60; 0.81]). Competence positively predicted effectiveness (*B* = 0.62, *SE* = 0.05, 95% CI [0.52; 0.72]) and liking (*B* = 0.49, *SE* = 0.04, 95% CI [0.41; 0.57]) which positively predicted leader promotability (effectiveness: *B* = 0.66, *SE* = 0.04, 95% CI [0.58; 0.74]; liking: *B* = 0.65, *SE* = 0.05, 95% CI [0.56; 0.74]). Dominance negatively predicted effectiveness (*B* = −0.66, *SE* = 0.06, 95% CI [−0.78; −0.55]) and liking (*B* = −0.72, *SE* = 0.05, 95% CI [−0.81; −0.63]) which positively predicted leader promotability (effectiveness: *B* = 0.66, *SE* = 0.04, 95% CI [0.58; 0.74]; liking: *B* = 0.74, *SE* = 0.05, 95% CI [0.64, 0.83]). Yet, the bootstrapping confidence intervals of the index of the moderated mediation included zero, which indicates that indirect effects did not differ according to leader gender (see Table 6 for the respective results).^14^ Thus, H3 to H4 were only partially supported.

**Table 6:** Indirect effects and index of the moderated serial mediation for H3 to H4.

Condition	Predictor	1st Mediator	2nd Mediator	Outcome	Path	*B*	*SE*	*95% CI*
Woman	Leadership behavior	Warmth	Effectiveness	Promotability	X→M1→M2→O	0.60	0.09	[0.44, 0.79]
Man	Leadership behavior	Warmth	Effectiveness	Promotability	X→M1→M2→O	0.63	0.09	[0.47, 0.80]
Index of moderated mediation						−0.03	0.05	[−0.13, 0.08]
Woman	Leadership behavior	Warmth	Liking	Promotability	X→M1→M2→O	0.75	0.10	[0.56, 0.97]
Man	Leadership behavior	Warmth	Liking	Promotability	X→M1→M2→O	0.79	0.10	[0.61, 1.00]
Index of moderated mediation						−0.04	0.07	[−0.17, 0.10]
Woman	Leadership behavior	Morality	Effectiveness	Promotability	X→M1→M2→O	0.46	0.08	[0.33, 0.63]
Man	Leadership behavior	Morality	Effectiveness	Promotability	X→M1→M2→O	0.52	0.07	[0.40, 0.67]
Index of moderated mediation						−0.06	0.07	[−0.19, 0.08]
Woman	Leadership behavior	Morality	Liking	Promotability	X→M1→M2→O	0.47	0.07	[0.34, 0.61]
Man	Leadership behavior	Morality	Liking	Promotability	X→M1→M2→O	0.53	0.07	[0.40, 0.67]
Index of moderated mediation						−0.06	0.07	[−0.19, 0.08]
Woman	Leadership behavior	Competence	Effectiveness	Promotability	X→M1→M2→O	0.26	0.06	[0.16, 0.38]
Man	Leadership behavior	Competence	Effectiveness	Promotability	X→M1→M2→O	0.26	0.06	[0.14, 0.37]
Index of moderated mediation:						0.01	0.07	[−0.13, 0.15]
Woman	Leadership behavior	Competence	Liking	Promotability	X→M1→M2→O	0.20	0.04	[0.12, 0.30]
Man	Leadership behavior	Competence	Liking	Promotability	X→M1→M2→O	0.20	0.05	[0.11, 0.29]
Index of moderated mediation						0.01	0.06	[−0.11, 0.12]
Woman	Leadership behavior	Dominance	Effectiveness	Promotability	X→M1→M2→O	0.64	0.08	[0.49, 0.81]
Man	Leadership behavior	Dominance	Effectiveness	Promotability	X→M1→M2→O	0.64	0.08	[0.50, 0.81]
Index of moderated mediation						−0.01	0.06	[−0.11, 0.11]
Woman	Leadership behavior	Dominance	Liking	Promotability	X→M1→M2→O	0.77	0.09	[0.62, 0.96]
Man	Leadership behavior	Dominance	Liking	Promotability	X→M1→M2→O	0.78	0.09	[0.62, 0.96]
Index of moderated mediation						−0.01	0.07	[−0.14, 0.12]

*N* = 454. The moderated mediations included the covariates rater age, rater gender, and raters’ managerial responsibility. The indirect effects and the index of the moderated mediation were computed using bootstrapping (5,000 resamples).

## Discussion

Leadership behavior matters for the evaluation of leaders and leaders’ careers. In this paper, we showed that servant and directive leadership are related to leaders’ promotability. In our study, servant leaders were perceived as more effective and likable, and thus ultimately more promotable than directive leaders. We examined the underlying mechanisms of this relationship. The facets of the leader’s perceived communion and agency explained the relationship between leadership behavior and these serial outcomes. Specifically, we found a *positive* relationship of servant (vs. directive) leadership with perceived leader effectiveness and liking. Servant leadership related to more effectiveness and liking via higher warmth, morality, and competence perceptions as well as via lower dominance perceptions. Warmth, morality, and competence positively related, whereas dominance negatively related to leader promotability via perceived leader effectiveness and liking. We also examined whether leader gender biases the evaluations of servant and directive leadership. As our results show, servant leadership was indeed more expected of women leaders. However, directive leadership was expected of women leaders and men leaders alike. This supports recent findings that an agentic leadership behavior is equally expected of both genders, while more communal leadership behavior is more expected of women (Hentschel et al., 2018). Contrary to our expectations, our results indicate no gender-biased evaluations of servant or directive leadership. Both women and men were perceived as equally communal and agentic for the same leadership behavior.

### Theoretical implications

Our results have implications for research on leader promotability regarding servant and directive leadership. We found that servant leaders were perceived as more effective, likable, and promotable than directive leaders, which aligns with previous research highlighting numerous positive outcomes of servant leadership for organizations and followers (Hoch et al., 2018; Eva et al., 2019; Lee et al., 2020). Up to now, it remained unclear whether servant leadership also serves leaders themselves. Our research provides evidence that also leaders themselves benefit from servant leadership. So far, servant leadership has been shown to be effective across cultures (Pekerti and Sendjaya, 2010; Zhang et al., 2021). We provide evidence that servant leadership is universally effective concerning gender as its evaluation does not vary depending on leader gender. In other words, servant leadership seems to benefit the careers of women and men alike.

Our research also highlights the relevance of examining gender-biased evaluations for each leadership behavior. We found no evaluative bonus or penalty for servant and directive leadership. Yet, research on other communal and agentic behaviors has at least partly demonstrated a bonus or penalty (e.g., Rudman et al., 2012; Hentschel et al., 2018). It could thus be misleading to generalize from one communal or agentic leadership behavior onto another. Regarding directive leadership, our findings are consistent with expectancy-violation theory (Jussim et al., 1987; Prentice and Carranza, 2004). Since directive leadership was equally expected of women and men, directive women leaders and directive men leaders received equal evaluations. This aligns with previous research showing no gender differences in expectation and evaluation of autocratic leadership, another agentic yet more strongly domineering leadership behavior (Hentschel et al., 2018). Our findings also resonate with research that women need to exhibit agency to prove that they have leadership qualities (Johnson et al., 2008; Bongiorno et al., 2014). According to our findings, directive women leaders seem to meet this agency expectation. Thus, they might have been perceived to own the same leadership abilities as men leaders.

An alternative explanation for our findings may be the selected leadership behaviors. Servant leaders were perceived to display a certain level of directive leadership. Raters may have assumed that servant leaders, by default, provide a certain degree of guidance and direction to followers, the minimal requirements for good leadership. Women and men showing directive leadership might have just been perceived as fulfilling the typical leader role. Consistently, according to the mean values across our four conditions, directive leaders were deemed as more prototypical than servant leaders. In contrast to directive leaders, servant leaders might have been perceived as exceptional leaders. They may have been assumed to exhibit the behaviors of directive leadership augmented by servant leadership. In this regard, servant leaders might positively violate expectations how leaders typically are and thus receive an evaluative bonus in line with expectancy-violation theory (Prentice and Carranza, 2004).

Even though servant leadership was expected more of women than of men, servant men leaders were not evaluated more favorably. This seems to contradict expectancy-violation theory (Prentice and Carranza, 2004). Yet, two different violations might have occurred for which this theory does not account. In its original conception, expectancy-violation theory focused on personal space violations (Burgoon, 1978, 2015). These could either be perceived as positive, resulting in a bonus, or as negative, resulting in a penalty. But leadership behavior may involve positive and negative violations at the same time. Servant men leaders may have gained no communion bonus due to a penalty for lacking dominance. Indeed, we found servant leaders to be perceived as low on dominance. The penalty for this dominance deficit may be evident in a devaluation of men. Servant leaders put their followers first and support others’ careers instead of their own career. Hence, servant men leaders might seem to violate prescriptions that men should be competitive and dominant (see also Prentice and Carranza, 2002; Rudman et al., 2012). As dominance is linked to status, men (but not women) who lack dominance seem to violate the gender hierarchy (for a discussion, see Moss-Racusin et al., 2010; Rudman et al., 2012). This aligns with previous research that found no bonus for servant men leaders related to follower outcomes (Lemoine and Blum, 2021). Our reasoning could also explain why men receive a communion bonus for transformational leadership (Hentschel et al., 2018). Instead of putting followers first, transformational leadership focuses on reaching organizational goals (Stone et al., 2004) and may not violate men’s dominance prescriptions. Concluding, the communion bonus for servant men leaders may have been obscured by a dominance deficit.

Our research further adds knowledge on the mechanisms underlying the evaluation of servant and directive leadership. We established a mediating role of perceived leader communion (i.e., warmth, morality) and agency (i.e., competence, dominance). Interestingly, our results do not align with shifting standards theory (Biernat, 2012). Women and men were perceived to score equally on communion and agency for the same leadership behavior and there was no gender-biased evaluation. Instead, our findings align with newer research suggesting competence to be perceived as equally characteristic of both genders (Hentschel et al., 2019; Eagly et al., 2020).

Focusing on the facets of communion and agency, our results underline the need to distinguish competence and dominance as subdimensions of agency (Rosette et al., 2016). Perceived leader competence was positively, and perceived leader dominance negatively, related to perceived leader effectiveness, liking, and promotability. Regarding the content of both agentic dimensions, competence seems to be socially desirable, whereas dominance is generally deemed undesirable (Prentice and Carranza, 2002; Ma et al., 2022).

### Managerial implications

Our results provide rich implications for managers and organizations that want to promote effective leadership behaviors, leaders’ careers, and gender equality. As our findings suggest, servant leaders are perceived as more effective, likable, and promotable compared to directive leaders. Thus, servant leadership holds benefits for leaders beyond the positive outcomes shown for organizations and followers (e.g., Hoch et al., 2018). Hence, leaders may be more motivated to show servant leadership if this leadership behavior also serves their careers. Importantly, women and men servant leaders profited equally from servant leadership. Thus, organizations are well-advised to provide leadership training on how to become a servant leader (e.g., training mindfulness; Pircher Verdorfer, 2016) to establish effective leadership behaviors and to advance women in leadership. Consistent with findings on the more important role of structural than individual adjustments (Gloor et al., 2020), servant leadership should be seen as a steppingstone on the way to changing the system toward gender equality. It would be short-sighted to promote servant leadership as a general cure to women’s underrepresentation or disadvantage in leadership.

Our findings offer recommendations for leaders on how to be seen as more effective and likable. In this regard, leaders benefit from being perceived as competent, warm, or moral. Being perceived as dominant harms their perceived leader effectiveness, liking, and thus their promotability. Hence, leaders are generally well advised to display competence, warmth, and morality and avoid dominant behaviors.

### Limitations and future research

Despite its contributions, our research holds several limitations. First, we implemented a fictional business case scenario in an online experimental environment rather than examining the evaluation of real supervisors’ leadership behavior. We used the conditions of the scenarios as independent variables and assumed that women and men who exhibit the same behavior are perceived as equally exhibiting servant leadership or directive leadership. The dependent variables were assessed by raters who were supposed to imagine being supervised by the depicted leader. Yet, the supervised followers seldom have a say in who is promoted in the organization. Moreover, our scenario contained no competitive context where raters could decide on whether either a woman or a man showing the same leadership behavior should be promoted as leader. Future research may examine the proposed relationships in the workplace and test the gender-biased evaluation of other leadership behaviors. This may also be conducted with raters as promotion committee members and in the field.

Second, we examined the evaluation of a formal supervisor. Raters might have been biased as the supervisor was assigned to her/his position with formal power instead of claiming the leader role herself/himself (see DeRue and Ashford, 2010). Claiming the leadership role might evoke perceptions of a will to lead. Claiming the leadership role might have a gendered impact on the leader’s perceived promotability as self-promoting harms women’s likability but not men’s (e.g., Rudman and Glick, 2001). On the other hand, if women leaders are granted the position of the leader by others, this might increase perceptions of their leadership competence. Thus, results might differ for a scenario about an informal or self-chosen leader.

Finally, we did not account for the intersectionality of gender and other discrimination variables like for example ethnicity or age via the provided silhouettes. Raters had probably primarily thought about White leaders as White people prevail in Western societies’ leadership positions (Alliance for Board Diversity Census, 2021). Nevertheless, these limitations hold options for future research. Due to the intersection of gender and ethnic stereotypes (e.g., Rosette et al., 2016), Asian women are stereotyped as very feminine and passive (Galinsky et al., 2013; Rosette et al., 2016), while Black men are stereotyped as very masculine and threatening (e.g., Galinsky et al., 2013; Todd et al., 2016). Thus, directive leadership may provide an agency bonus for Asian women leaders and servant leadership may provide a communion bonus for Black men leaders compared to their ethnic counterparts. Age might also play a significant role. Our silhouettes were rated as belonging to adults in their mid-thirties.^15^ Younger leaders are seen as less effective and likable compared to middle-aged leaders and leader age can even overshadow gender information (Daldrop et al., 2023). It remains to be seen whether younger leaders profit more from implementing servant leadership compared to middle-aged leaders thanks to a communion bonus, or whether they suffer due to losing status (see Buengeler et al., 2016). Despite being universally effective across cultures (Pekerti and Sendjaya, 2010), servant women leaders and servant men leaders could receive differing evaluations depending on the local culture, gender expectations, and strictness of gender roles (e.g., gender egalitarianism, House et al., 2004). Hence, it would be interesting to examine the leadership evaluations with raters’ individual cultural orientations as moderators.

For future research on expectancy violation, we encourage to first examine whether the expectations for a certain leadership behavior are biased. If the same leadership behavior for example is expected of women and men, then no expectancy violation and no evaluative bias can occur. Thus, we welcome research regarding gender-biased and intersectional leader prototypes of women leaders and men leaders. In addition, the valence (positive vs. negative) of the respective behavior should also be examined. Based on the valence, one can conclude whether an expectancy violation is perceived as positive or negative, resulting in either bonus or penalty.

Future research should further examine which role a leader’s perceived communion and agency play in promotion decisions. A person’s perceived communion contributes more to forming a first overall judgment than a person’s perceived agency, but the importance of agency increases with increasing outcome dependency (Abele and Wojciszke, 2007). Thus, a leader’s communion and agency may play a pivotal role in leadership evaluations as well as in resulting promotion decisions, depending on how well the promotion committee knows the leader. Future research should address this relationship and the role of rater’s perceived outcome dependency which rater’s gender stereotypes could influence.

Despite servant leadership’s seeming gender-neutral evaluation, we encourage future research regarding the interplay with leader gender. For example, Eva et al. (2019) suggested examining whether leaders are negatively influenced by exhibiting servant leadership as evident in higher stress or burn-out. Women leaders could have higher emotional costs for displaying servant leadership than men because they still primarily fulfill the role of a “servant” in house- and care-work. Due to gender roles of women as caretakers, women might receive less appreciation for displaying servant leadership compared to men. This lower appreciation would reduce the resources that servant leaders gain from helping their followers, as appreciation seems to compensate for the resources lost by showing servant leadership (Xu and Wang, 2018). If women leaders are not internally motivated to practice servant leadership but receive external pressure to do so, they will likely suffer physically and psychologically (Vial and Cowgill, 2022). Thus, it will be important to examine potential gender differences in servant leadership’s effects on leaders.

## Conclusion

In this paper, we examined whether leaders themselves profit more from implementing servant or directive leadership behavior – and if leader gender plays a role in this. Our research suggests that servant leaders are perceived to be more effective, likable, and promotable than directive leaders, regardless of leader gender. Being seen as warm, moral, and competent is positively, and being seen as dominant is negatively, related to perceived leader effectiveness, liking, and thus promotability. Leadership behaviors are key to leadership development and strategies to empower aspiring (women) leaders. As servant leadership seems to be gender-neutral in its evaluation, we suggest servant leadership as a leadership behavior that serves not only organizations and followers but also benefits leaders’ careers.

## Data availability statement

The raw data supporting the conclusions of this article will be made available by the authors, without undue reservation.

## Author contributions

AB primarily developed, designed, and analyzed the study and wrote the first draft. CB contributed to all steps of the process. All authors contributed to the article and approved the submitted version.

## Footnotes

^1^We use the term “gender-biased” in combination with evaluation when referring to different evaluations of women and men as leaders. We do not refer to differences in the evaluation behavior of women or men who rate these leaders.

^2^In line with Van Knippenberg and Van Knippenberg (2005), we use the term leader prototypicality referring to “the leader’s representativeness of a leader category (i.e., matching the stereotype of a leader)” (p. 27) as described in leadership-categorization theory. To avoid confusion, we distinguish this conceptualization of leader prototypicality from the concept of group prototypicality that is used in social-identity analysis and refers to a leader’s “representativeness of the work group, team, or organization that the leader is leading” (Van Knippenberg and Van Knippenberg, 2005, p. 27). We concentrate on prototypicality concerning typical leaders (Lord et al., 1984; Barsalou, 1985; Junker and Van Dick, 2014; Van Quaquebeke et al., 2014) because compared to the ideal leader prototype, the typical leader prototype includes more ineffective characteristics and thus reflects the general image of a leader (Schyns and Schilling, 2011). In addition, the typical leader prototype comprises more agentic characteristics (Epitropaki and Martin, 2004; Offermann and Coats, 2018) and raters believe that, first and foremost, agency is related to the leader role (Eagly and Karau, 2002).

^3^The reversed item was excluded because the corrected item-total correlation was below 0.30 and Cronbach’s alpha was better when the item was deleted (Field, 2018; see Supplementary material, section #1.2).

^4^For the prescriptive servant and directive leadership condition, the Shapiro–Wilk test indicated that the differences between the scores for women and men were non-normally distributed (*p* < 0.001) which is negligible as our sample size exceeded *n* = 30 (Stone, 2010). Outliers remained in the analyses as results did not differ when they were excluded.

^5^We recruited participants via the acquisition platform *Respondi* in exchange for a participation fee. Pre-selection criteria for our online survey included age and working hours per week to ensure that participants are aged 18 to 67 years and are full-time employed at an organization or public service and thus are used to organizational hierarchies.

^6^To prevent careless responding, we applied recommendations for prevention and precaution of Goldammer et al. (2020); e.g., payment for participation, providing personal instructions. Initially, we collected data from 460 persons who completed the full questionnaire. At the end of the survey, we asked participants to indicate whether they filled out the questionnaire sincerely so that their data can be used for statistical analyses, with no consequences for their reward. Six participants indicated that they were just clicking through and were excluded from the analyses (Aust et al., 2013).

^7^The results of the hypotheses did not differ when the 31 participants were excluded that indicated a difficult or very difficult imagination of the scenario.

^8^We discuss the potential of demand effects for our measurement of gender role beliefs in the Supplementary material (section #3.2.2) because these manipulation checks took place before participants had to answer the scale about gender role beliefs. We also computed the analyses of H1 to H4 for the sample that included participants who failed the manipulation checks (*N* = 740). The results for the hypotheses tests did not differ.

^9^Hentschel et al. (2018) excluded participants who failed to have a mean difference of one scale point in the direction of the intended leadership behavior. If we had followed their procedure, our sample would have been reduced to *N* = 337. To secure the power of our analyses, we calculated the results with the larger sample. A servant leader may be perceived as giving task-directed orders and clearly formulating expectations, despite these behaviors not being mentioned in the scenario. As all participants had correctly answered the general manipulation check regarding the described leadership behavior, they should have understood the manipulation as intended. However, we conducted all analyses with the smaller sample. The results of the hypotheses tests did not differ.

^10^We conducted a confirmatory factor analysis in *R* with *lavaan* to examine whether the measured variables represent distinct concepts. The results for our hypothesized 9-factor model suggest a good fit with the data, *χ*^2^(1733, 454) = 4082.54, *p* < 0.001, CFI = 0.92, TLI = 0.91, RMSEA = 0.06, SRMR = 0.06, compared to a single factor model, *χ*^2^(1769, 454) = 13900.92, *p* < 0.001, CFI = 0.57, TLI = 0.55, RMSEA = 0.12, SRMR = 0.15, Δ*χ*^2^(36) = 9818.38, *p* < 0.001. An eight-factor model merging perceived warmth and morality into the factor communion, *χ*^2^(1741, 454) = 4453.30, *p* < 0.001, CFI = 0.90, TLI = 0.90, RMSEA = 0.06, SRMR = 0.06, Δ*χ*^2^(8) = 370.77, *p* < 0.001, as well as a seven-factor model merging perceived competence and dominance into the factor agency, *χ*^2^(1748, 454) = 5579.64, *p* < 0.001, CFI = 0.86, TLI = 0.86, RMSEA = 0.07, SRMR = 0.07, Δ*χ*^2^(15) = 1497.10, *p* < 0.001, showed a worse fit with the data.

^11^Before testing H1, we conducted a multivariate analysis of covariance (MANCOVA) to test the effects of the leadership behavior, leader gender, and their interaction on all four dependent variables including the covariates. The MANCOVA revealed a statistically significant difference between the leadership behaviors on the dependent variables, *F*(4, 444) = 128.49, *p* < 0.001, partial *η*^2^ = 0.54, Wilk’s Λ = 0.46. However, we found no significant main effect of leader gender, *F*(4, 444) = 1.45, *p* = 0.22, partial *η*^2^ = 0.01, Wilk’s Λ = 0.99, and no significant interaction effect between leadership behavior and leader gender on the combined dependent variables, *F*(4, 444) = 0.79, *p* = 0.53, partial *η*^2^ = 0.01, Wilk’s Λ = 0.99.

^12^The residuals of perceived leader effectiveness and liking were normally distributed, as determined by the Shapiro–Wilk test, (*p* > 0.05). The residuals of perceived leader promotability were normally distributed when assessed by the Kolmogorov–Smirnov test, (*p* > 0.05), but they were not normally distributed as determined by the stricter Shapiro–Wilk test (*p* = 0.03). This is another reason why we conducted bootstrapping.

^13^For perceived leader prototypicality the homogeneity of regression slopes was violated for the interaction term of the independent variable leadership behavior and the covariate rater gender, as the interaction terms were statistically significant (*p* < 0.05).

^14^As additional analyses we computed ANCOVAs to examine the main effects of leadership behavior and leader gender and their interaction on the facets of communion and agency. Results revealed that servant leaders compared to directive leaders received significantly higher ratings of warmth (*F*(1, 447) = 498.88, *p* < 0.001, partial *η*^2^ = 0.53), morality (*F*(1, 447) = 210.70, *p* < 0.001, partial *η*^2^ = 0.32), competence (*F*(1, 447) = 50.57, *p* < 0.001, partial *η*^2^ = 0.10), and lower ratings of dominance (*F*(1, 447) = 523.49, *p* < 0.001, partial *η*^2^ = 0.54). We computed pairwise comparisons and used the robust method bootstrapping (1,000 resamples, Field, 2018). Pairwise comparisons revealed that servant leaders were perceived as warmer (*M_SL_* = 4.17, *M_DL_* = 2.44, 95% CI [1.58, 1.88], *p* < 0.01), more moral (*M*_SL_ = 4.25, *M*_DL_ = 3.14, 95% CI [0.97, 1.26], *p* < 0.01), more competent (*M*_SL_ = 4.03, *M*_DL_ = 3.46, 95% CI [0.42, 0.73], *p* < 0.01) and less dominant (*M*_SL_ = 1.76, *M*_DL_ = 3.42, 95% CI [−1.82, −1.53], *p* < 0.01) than directive leaders. We again found no main effect for leader gender and no interaction effect between leadership behavior and leader gender.

^15^Interestingly, servant leaders were perceived as younger than directive leaders (see Supplementary material, section #3.2).
