# Supplementary material for: Servant versus directive leadership and promotability: does leader gender matter?
Source: Front Psychol. 2023 Dec 11;14:957121. doi: 10.3389/fpsyg.2023.957121 (PMC10749661; doi:10.3389/fpsyg.2023.957121)
Supplement: Supplementary file 1 [file Data_Sheet_1.docx]

Supplementary Material

**Supplementary Table of Contents**

1. **Pre-Study 1**

1.1 Sample Characteristics

1.2 Operationalization

1. **Pre-Study 2**

2.1 Sample Characteristics

2.2 Operationalization

2.3 Supplementary Results

1. **Main-Study**

3.1 Manipulation

3.2 Supplementary Results

3.2.1 Assertiveness

3.2.2 Raters’ Gender Role Beliefs

1. **Supplementary References**

# Pre-Study 1

We conducted a pre-study to test which leadership behavior is expected of women and men. For this, we contrasted servant leadership with directive leadership. We recruited an online sample and randomly assigned participants to two conditions, *typical* or *ideal* leadership expectations.

## Sample Characteristics

Initially, our overall sample consisted of *N* = 100 participants. Two participants indicated that they were just clicking through and were thus excluded from the analyses (Aust et al., 2013). We also excluded six respondents who failed the attention checks. This resulted in our overall sample of *N* = 92 participants.

The sample of the *typical* condition consisted of 44 participants (70.5% female, 2 missing values) with an average age of 28.95 years (*SD* = 8.97). 16 participants were working full-time (36.40%), 15 were working part-time (34.1%) and 12 persons were not working at all (27.3%, 1 missing value). Seven participants held a leadership position (15.9%, 1 missing value). Concerning their education, 29 participants held a university degree (65.9%), eight participants had an advanced school leaving certificate (18.2%), five participants had an intermediate school leaving certificate (11.4%), and one participant had a PhD degree (2.3%, 1 missing value).

The sample of the *ideal* condition comprised 48 participants (77.1% female) with an average age of 28.65 years (*SD* = 9.82). 19 participants were not working at all (39.6%), 15 participants were working part-time (31.3%), and 14 participants were working full-time (29.2%). Additionally, six participants held a leadership position (12.5%, 1 missing value). Concerning their education, 29 participants had a university degree (60.4%), 17 participants held an advanced school leaving certificate (35.4%), one participant had an intermediate school leaving certificate (2.1%), and one participant had a PhD degree (2.1%).

## Operationalization

Participants indicated on a 7-point Likert scale ranging from (1) *do not agree at all* to (7) *totally agree* how much they expected a woman or man as supervisor to typically/ideally exhibit the leadership behaviors that were presented in a randomized order with a distractor task in-between. The distractor task was as follows: “You have 60 seconds to name as many consequences of this unimaginable event as you can: "What would happen if all the mice in the world disappeared?"” (German original: Sie haben 60 Sekunden Zeit, um so viele Folgen dieses unvorstellbaren Ereignisses wie möglich zu nennen: "Was würde passieren, wenn alle Mäuse auf der Welt verschwinden würden?").

*Servant leadership* was operationalized by the seven items of the SL-7 (Liden et al., 2015) with one item being adapted to “I can seek help from her/him if I have a personal problem.” (adapted by using *can* instead of *could* and *have* instead of *had*). The German translation was taken from Ruthus (2019). The reliabilities were computed for the ideal condition (α_woman_ = 0.68, α_man_ = 0.83) and for the typical condition (α_woman_ = 0.82, α_man_ = 0.80).

*Directive leadership* was measured by the five items taken from Northouse (2016). The reliabilities were computed for the ideal condition (α_woman_ = 0.78, α_man_ = 0.66) and for the typical condition with four items (α_woman_ = 0.81, α_man_ = 0.83; the reversed item was excluded from the analyses for both conditions because its corrected item-total correlation for the typical condition was far below 0.30 and Cronbach’s alpha was better when the item was deleted; Field, 2018). Supplementary Table 1 shows the instructions for each condition in German and the English translation^[[1]](#footnote-1)^.

**Supplementary Table 1.** Instructions regarding the typical or ideal leadership behavior expected of women leaders and men leaders.

| **Condition** | **German Original** | **English Translation** |
| --- | --- | --- |
| **Typical/Woman** | Im Folgenden geht es um Ihre Erwartungen bezüglich typischen Verhaltens.  Bitte stellen Sie sich vor, eine *Frau* als formelle Vorgesetzte zu haben.  Welches Führungsverhalten zeigt eine *Frau* *typischerweise*? | The following refers to your expectations regarding typical behavior.  Please imagine having a *woman* as your formal supervisor.  What leadership behavior does a *woman typically* exhibit? |
| **Typical/Man** | Im Folgenden geht es um Ihre Erwartungen bezüglich typischen Verhaltens.  Bitte stellen Sie sich vor, einen *Mann* als formellen Vorgesetzten zu haben.  Welches Führungsverhalten zeigt ein *Mann typischerweise*? | The following refers to your expectations regarding typical behavior.  Please imagine having a *man* as your formal supervisor.  What leadership behavior does a *man typically* exhibit? |
| **Ideal/Woman** | Im Folgenden geht es um Ihre Erwartungen bezüglich idealen Verhaltens.  Bitte stellen Sie sich vor, eine *Frau* als formelle Vorgesetzte zu haben.  Welches Führungsverhalten zeigt eine *Frau idealerweise*? | The following refers to your expectations regarding ideal behavior.  Please imagine having a *woman* as your formal supervisor.  What leadership behavior does a *woman ideally* exhibit? |
| **Ideal/Man** | Im Folgenden geht es um Ihre Erwartungen bezüglich idealen Verhaltens.  Bitte stellen Sie sich vor, einen *Mann* als formellen Vorgesetzten zu haben.  Welches Führungsverhalten zeigt ein *Mann idealerweise*? | The following refers to your expectations regarding ideal behavior.  Please imagine having a *man* as your formal supervisor.  What leadership behavior does a *man ideally* exhibit? |

In addition to the demographics, we asked participants whether they had thought of their (formal) supervisor while answering the questionnaire. We also included one attention check into each of the questionnaires about the typical/ideal leadership behaviors namely: “I am attentive and click (7/1) strongly dis/agree.”. Next, participants indicated their gender role beliefs by responding to the 20-items of an adapted version of Larsen and Long’s (1988) *Attitudes toward Sex Roles* *Scale*. Hereby, we wanted to pre-examine the internal consistency of this scale for the main study. In this scale we included an attention check, too. Finally, participants responded to a sincerity check whether their data should be used for statistical analyses.

# Pre-Study 2

We conducted another pre-study to validate the stimulus material that we planned to use in the main study to increase gender salience of the depicted leader. The stimulus material consisted of either silhouettes or computer-generated and aged pictures of a woman and a man.

## Sample Characteristics

We recruited a sample of *N* = 42 participants online in Germany (52.4 % female, 38.1% male, 4.8% diverse, 4.8% did not specified their gender) with a mean age of 38.27 years (*SD* = 14.62, 1 missing value). Regarding their current occupation, 24 participants were employed at an organization (57.1%), 12 participants indicated to go to university (28.6%), and six participants indicated having another occupation (14.3%). Concerning their education, 19 participants held a university degree (45.2%), 12 participants had an advanced school leaving certificate (28.6%), six participants had an intermediate school leaving certificate (14.3%), three participants indicated to have another educational degree (7.1%) and two participants had a PhD degree (4.8%).

## Operationalization

Participants were presented with a female and a male person represented by a silhouette (Hernandez Bark et al., 2022) or a face (see Supplementary Figure 1) in a randomized order with a distractor task between the faces and the silhouettes. We adapted the silhouette of the woman as we presented it without the grey and black layers in the background by clearly cutting out the silhouette. We generated the two faces via merging pictures taken from the *Face Research Lab London Set* (DeBruine & Jones, 2017) using the tool *WebMorph* (DeBruine, 2018). The resulting faces were aged by the software *Photoshop* because adults in their 40s match the leadership prototype better compared to younger adults (Buengeler et al., 2016). The instruction below the presented stimulus was: “Please take a close look at the silhouette/face presented. Take a moment to do so.” (German original: Bitte schauen Sie sich die präsentierte Silhouette/das präsentierte Gesicht genau an. Nehmen Sie sich einen Moment Zeit dafür.) On the next page, we instructed the participants as follows: “Please answer the following questions about the person whose silhouette/face you see spontaneously and from your gut. We are interested in your first impression.” (German Original: Bitte beantworten Sie die folgenden Fragen zu der Person, deren Silhouette/Gesicht Sie sehen, spontan und aus dem Bauch heraus. Wir sind an Ihrem ersten Eindruck interessiert). The respective silhouette or face was again presented below the instruction.

The distractor task in-between was as follows: “For an unimaginable event given below, you are to think of as many consequences as possible. Do not give reasons or complicated explanations. Write down your ideas line by line as bullet points. You have 60 seconds to name as many consequences of this unimaginable event as you can: "What would happen if all the mice in the world disappeared?"” (German original: Für ein unvorstellbares Ereignis, das unten angegeben ist, sollen Sie sich so viele Folgen wie möglich ausdenken. Geben Sie keine Gründe oder komplizierte Erklärungen an. Schreiben Sie Ihre Ideen Zeile für Zeile als Stichpunkte auf. Sie haben 60 Sekunden Zeit, um so viele Folgen dieses unvorstellbaren Ereignisses wie möglich zu nennen: "Was würde passieren, wenn alle Mäuse auf der Welt verschwinden würden?").


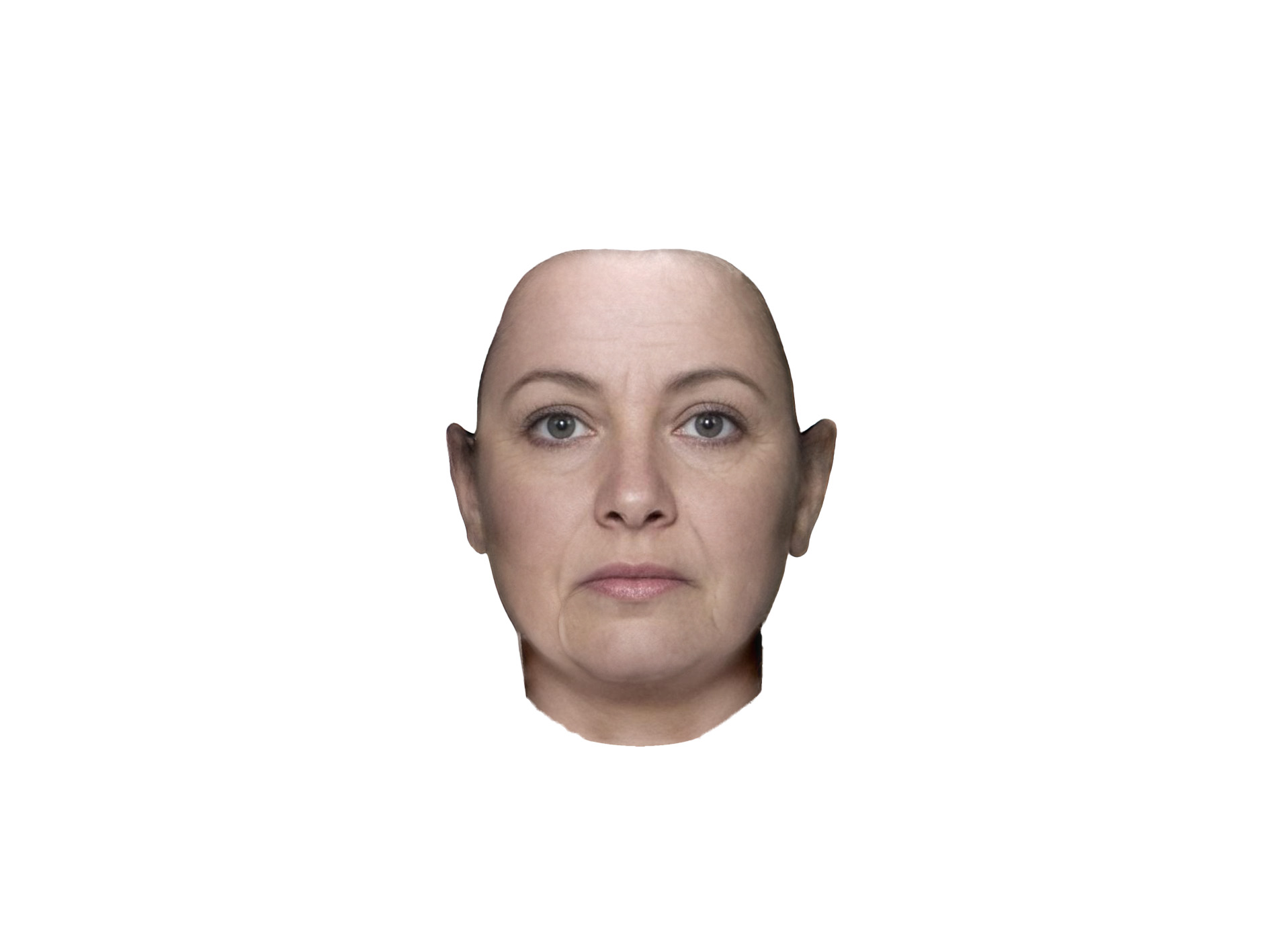

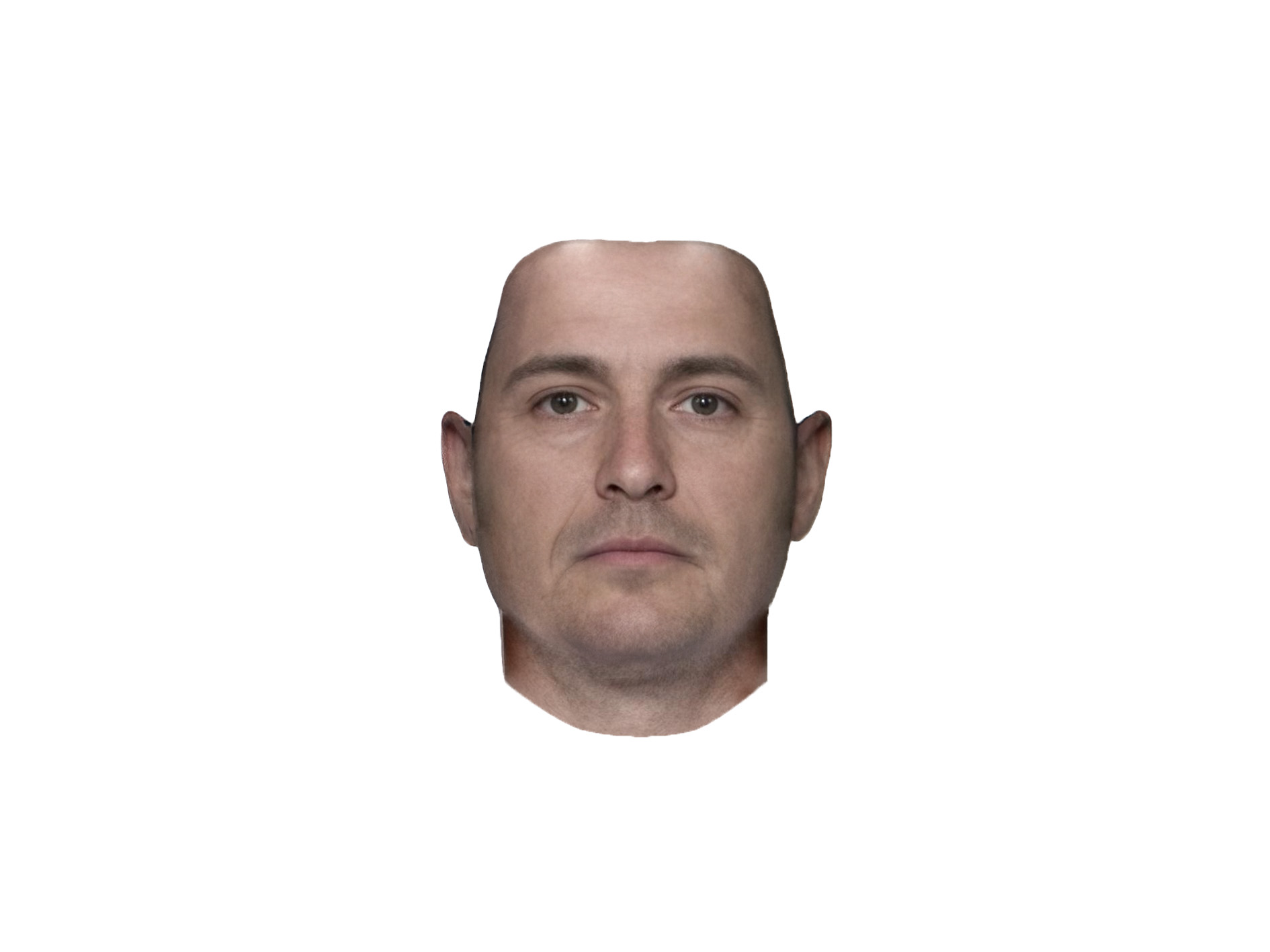


**Supplementary Figure 1.** Aged faces of a woman and a man.

Regarding the presented stimuli, participants indicated on a 5-point Likert scale (from 1 to 5, for exact wording of scale anchors see Supplementary Table 2) the depicted person’s perceived attractiveness, perceived intelligence, perceived liking, perceived dominance, perceived competence, perceived warmth, and perceived morality. They also indicated how friendly they would treat the person and how much they would be interested to meet the person. Additionally, they indicated the perceived age and perceived gender of the presented silhouette. The questions were created by the authors, were presented in a randomized order, and are listed in Supplementary Table 2. The instruction at the beginning of the survey was as follows: “Below you will see four images. Two represent faces and two abstract silhouettes of people. Please look carefully at the pictures presented. Afterwards, you will be asked some questions about them. Please answer spontaneously. There is no right or wrong. We are interested in your first impression.” (German original: Im Folgenden werden Ihnen vier Bilder präsentiert. Zwei stellen Gesichter und zwei abstrakte Silhouetten von Personen dar. Bitte schauen Sie sich die präsentierten Bilder genau an. Anschließend werden Ihnen einige Fragen dazu gestellt. Bitte antworten Sie spontan. Es gibt kein richtig oder falsch. Wir sind an Ihrem ersten Eindruck interessiert.)

## Supplementary Results

The residuals were in neither case normally distributed. Outliers remained in the analyses as results did not differ when excluded. The differences of none of the variables were normally distributed as assessed by the Shapiro-Wilk test (*p* < .05), yet this is negligible as our sample size exceeded *n* = 30 (Stone, 2010). The means and standard deviations are depicted in Supplementary Table 3.

**Supplementary Table 2.** Items of pre-study 2.

| **Construct** | **German Original** | **English Translation** |
| --- | --- | --- |
| **Perceived** **Age** | Für wie alt schätzen Sie diese Person? | How old do you think this person is? |
| **Perceived** **Attractiveness** | Für wie attraktiv halten Sie diese Person? Bitte berücksichtigen Sie nur die aussehensbezogene Attraktivität.  (1) sehr unattraktiv  (2) unattraktiv  (3) neutral  (4) attraktiv  (5) sehr attraktiv | How attractive do you think this person is? Please consider only the appearance-related attractiveness.  (1) very unattractive  (2) unattractive  (3) neutral  (4) attractive  (5) very attractive |
| **Perceived** **Intelligence** | Für wie intelligent halten Sie diese Person?  (1) sehr unintelligent  (2) unintelligent  (3) neutral  (4) intelligent  (5) sehr intelligent | How intelligent do you think this person is?  (1) very unintelligent  (2) unintelligent  (3) neutral  (4) intelligent  (5) very intelligent |
| **Perceived** **Liking** | Basierend auf Ihrem ersten Eindruck, inwieweit würden Sie diese Person mögen oder nicht mögen?  (1) überhaupt nicht mögen  (2) nicht mögen  (3) neutral  (4) mögen  (5) sehr mögen | Based on your first impression, to what extent would you like or dislike this person?  (1) do not like at all  (2) dislike  (3) neutral  (4) like  (5) like very much |
| **Interest in Getting to Know the Person** | Basierend auf Ihrem ersten Eindruck, wie interessiert wären Sie daran, diese Person kennenzulernen?  (1) gar nicht interessiert  (2) nicht interessiert  (3) neutral  (4) interessiert  (5) sehr interessiert | Based on your first impression, how interested would you be to get to know this person?  (1) not interested at all  (2) not interested  (3) neutral  (4) interested  (5) very interested |
| **Treating the Person in a Friendly Manner** | Wie würden Sie diese Person aufgrund Ihres ersten Eindrucks behandeln?  (1) sehr unfreundlich  (2) unfreundlich  (3) neutral  (4) freundlich  (5) sehr freundlich | Based on your first impression, how would you treat this person?  (1) very unfriendly  (2) unfriendly  (3) neutral  (4) friendly  (5) very friendly |
| **Perceived** **Dominance** | Für wie dominant halten Sie diese Person?  (1) gar nicht dominant  (2) nicht dominant  (3) neutral  (4) dominant  (5) sehr dominant | How dominant do you think this person is?  (1) not dominant at all  (2) not dominant  (3) neutral  (4) dominant  (5) very dominant |
| **Perceived** **Competence** | Für wie kompetent halten Sie diese Person?  (1) sehr inkompetent  (2) inkompetent  (3) neutral  (4) kompetent  (5) sehr kompetent | How competent do you think this person is?  (1) very incompetent  (2) incompetent  (3) neutral  (4) competent  (5) very competent |
| **Perceived** **Warmth** | Für wie warmherzig halten Sie diese Person?  (1) gar nicht warmherzig  (2) nicht warmherzig  (3) neutral  (4) warmherzig  (5) sehr warmherzig | How warm do you think this person is?  (1) not warm at all  (2) not warm  (3) neutral  (4) warm  (5) very warm |
| **Perceived** **Morality** | Für wie integer halten Sie diese Person?  (1) gar nicht integer  (2) nicht integer  (3) neutral  (4) integer  (5) sehr integer | How much integrity do you think this person has?  (1) not integer at all  (2) not integer  (3) neutral  (4) integer  (5) very integer |
| **Perceived** **Gender** | Welches Geschlecht hat die dargestellte Person?  O Weiblich  O Männlich  O Divers  O Ich weiß nicht | What is the gender of the person depicted?  O Female  O Male  O Diverse  O I do not know |

**Supplementary Table 3.** Means and standard deviations for female and male faces and silhouettes.

| **Measures** | **Condition** | | | | |
| --- | --- | --- | --- | --- | --- |
|  | Gender | Face | | Silhouette | |
|  |  | *M* | *SD* | *M* | *SD* |
| Perceived Age | Female | 44.83 | 4.98 | 36.86^a^ | 6.20 |
|  | Male | 42.24 | 4.34 | 35.65^a^ | 5.24 |
|  |  |  |  |  |  |
| Perceived Attractiveness | Female | 2.90 | 0.73 | 3.45 | 0.63 |
|  | Male | 2.57 | 0.70 | 3.40 | 0.73 |
|  |  |  |  |  |  |
| Perceived Intelligence | Female | 3.50 | 0.60 | 3.67 | 0.65 |
|  | Male | 3.17 | 0.62 | 3.62 | 0.54 |
|  |  |  |  |  |  |
| Perceived Liking | Female | 2.83 | 0.70 | 3.17 | 0.49 |
|  | Male | 2.86 | 0.65 | 3.00 | 0.63 |
|  |  |  |  |  |  |
| Interest in Getting to Know the Person | Female | 2.57 | 0.83 | 3.12 | 0.74 |
|  | Male | 2.48 | 0.83 | 2.81 | 0.92 |
|  |  |  |  |  |  |
| Treating the Person in a Friendly Manner | Female | 3.40 | 0.70 | 3.69 | 0.47 |
|  | Male | 3.45 | 0.59 | 3.64 | 0.49 |
|  |  |  |  |  |  |
| Perceived Dominance | Female | 3.55 | 0.74 | 3.26 | 0.63 |
|  | Male | 3.31 | 0.84 | 3.62 | 0.62 |
|  |  |  |  |  |  |
| Perceived Competence | Female | 3.52 | 0.59 | 3.69 | 0.64 |
|  | Male | 3.21 | 0.61 | 3.60 | 0.73 |
|  |  |  |  |  |  |
| Perceived Warmth | Female | 2.79 | 0.75 | 3.02 | 0.52 |
|  | Male | 2.76 | 0.69 | 2.69 | 0.64 |
|  |  |  |  |  |  |
| Perceived Morality | Female | 3.21 | 0.68 | 3.21 | 0.68 |
|  | Male | 2.88 | 0.63 | 2.98 | 0.72 |
|  |  |  |  |  |  |
| Perceived Gender | Female | 1.02 | 0.15 | 1.10 | 0.37 |
|  | Male | 0.00 | 0.00 | 0.29 | 0.71 |

*Note. N* = 42, ^a^ *N* = 37.

Concerning the faces, the paired t-tests indicated that the female face was perceived as more attractive compared to the male face (*t*(41) = 2.55, *p* < .05), more intelligent (*t*(41) = 3.15, *p* < .01), more competent *t*(41) = 2.57, *p* < .05) and more moral *t*(41) = 2.65, *p* < .05).Yet, there were no differences regarding perceived liking (*t*(41) = -0.18, *p* = .86), interest in getting to know the person (*t*(41) = 0.64, *p* = .52), treating the person in a friendly manner (*t*(41) = -0.37, *p* = .71), perceived dominance (*t*(41) = 1.43, *p* = .16), and perceived warmth (*t*(41) = 0.18, *p* = .86). The female and male face significantly differed regarding their perceived gender (woman = 1, man = 0, diverse = 2, *t*(41) = 43.000, *p* < .001). Yet, both faces differed in their perceived age (*t*(41) = 3.65, *p* < .01) with the female face being perceived as older than the male face.

# Main Study

In the following, we report the written scenarios for each condition of our manipulation. We also report the supplementary results including the various confirmatory analyses, the full correlation table, the exploratory findings for leader assertiveness, and the results of the serial moderated mediation analyses with raters’ gender role beliefs (H5).

- 1. **Manipulation**

The manipulation was executed through the written scenario of each condition. Two independent variables were considered: leadership behavior via directive leadership (DL) and servant leadership (SL) as well as leader’s binary gender. Implementing a 2x2 design (leadership behavior [DL, SL] x leader gender [woman, man]), four different conditions derived. The scenarios were written in German. Prior to the scenarios, participants read the instruction: “The following text describes a situation in the workplace. Please read the text on the next page carefully. It is important that you put yourself in the scenario described. Please imagine that the person described is your formal supervisor in real life.” (German original: “Im folgenden Text wird eine Situation am Arbeitsplatz beschrieben. Bitte lesen Sie den Text auf der nächsten Seite aufmerksam durch. Es ist wichtig, dass Sie sich in das beschriebene Szenario hineinversetzen. Bitte stellen Sie sich vor, dass die beschriebene Person Ihnen im wahren Leben formell vorgesetzt ist.”). The German original and the English translation of the scenarios are depicted in Supplementary Table 4. The German original and the English translation of the instrumental manipulation check items are depicted in Supplementary Table 5.

**Supplementary Table 4.** Written scenarios for the manipulation of leadership behavior and leader gender.

| **Condition** | **German Original** | **English Translation** |
| --- | --- | --- |
| **DL/Woman** | Ihre Vorgesetzte lässt Sie wissen, was von Ihnen erwartet wird.  Ihre Vorgesetzte sagt Ihnen, was getan werden muss und wie es getan werden muss.  Ihre Vorgesetzte bittet Sie, die Standardregeln und Vorschriften zu befolgen.  Sie macht jedem und jeder ihrer Untergebenen deutlich, welche Rolle er oder sie in der Gruppe spielt.  Ihre Vorgesetzte erklärt das Leistungsniveau, das sie von Ihnen erwartet.  Sie würde niemals nur vage erklären, was von Ihnen bei der Arbeit erwartet wird. | Your supervisor lets you know what is expected of you.  Your supervisor tells you what needs to be done and how it needs to be done.  Your supervisor asks you to follow standard rules and regulations.  She makes it clear to each of her subordinates what his or her role is in the group.  Your supervisor explains the level of performance she expects from you.  She would never give vague explanations about what is expected of you on the job. |
| **DL/Man** | Ihr Vorgesetzter lässt Sie wissen, was von Ihnen erwartet wird.  Ihr Vorgesetzter sagt Ihnen, was getan werden muss und wie es getan werden muss.  Ihr Vorgesetzter bittet Sie, die Standardregeln und Vorschriften zu befolgen.  Er macht jedem und jeder seiner Untergebenen deutlich, welche Rolle er oder sie in der Gruppe spielt.  Ihr Vorgesetzter erklärt das Leistungsniveau, das er von Ihnen erwartet.  Er würde niemals nur vage erklären, was von Ihnen bei der Arbeit erwartet wird. | Your supervisor lets you know what is expected of you.  Your supervisor tells you what needs to be done and how it needs to be done.  Your supervisor asks you to follow standard rules and regulations.  He makes it clear to each of his subordinates what his or her role is in the group.  Your supervisor explains the level of performance he expects from you.  He would never give vague explanations about what is expected of you on the job. |
| **SL/Woman** | Ihre Vorgesetzte macht Ihre Karriereentwicklung zur Priorität.  Ihre Vorgesetzte betont, wie wichtig es ist, der Gemeinschaft etwas zurückzugeben, und stellt die Interessen ihrer Untergebenen über ihre eigenen.  Wenn Sie ein persönliches Problem haben, können Sie bei ihr Hilfe suchen.  Ihre Vorgesetzte erkennt, wenn bei Ihnen etwas Arbeitsbezogenes schiefläuft.  Ihre Vorgesetzte gibt Ihnen die Freiheit, mit schwierigen Situationen so umzugehen, wie Sie es für richtig halten.  Sie würde ethische Grundsätze nicht missachten, um erfolgreich zu sein. | Your supervisor makes your career development a priority.  Your supervisor emphasizes the importance of giving back to the community and puts the interests of her subordinates above her own.  If you have a personal problem, you can seek help from her.  Your supervisor recognizes when something work-related is going wrong.  Your supervisor gives you the freedom to handle difficult situations in the way that you feel is best.  She would not compromise ethical principles in order to achieve success. |
| **SL/Man** | Ihr Vorgesetzter macht Ihre Karriereentwicklung zur Priorität.  Ihr Vorgesetzter betont, wie wichtig es ist, der Gemeinschaft etwas zurückzugeben, und stellt die Interessen seiner Untergebenen über seine eigenen.  Wenn Sie ein persönliches Problem haben, können Sie bei ihm Hilfe suchen.  Ihr Vorgesetzter erkennt, wenn bei Ihnen etwas Arbeitsbezogenes schiefläuft.  Ihr Vorgesetzter gibt Ihnen die Freiheit, mit schwierigen Situationen so umzugehen, wie Sie es für richtig halten.  Er würde ethische Grundsätze nicht missachten, um erfolgreich zu sein. | Your supervisor makes your career development a priority.  Your supervisor emphasizes the importance of giving back to the community and puts the interests of his subordinates above his own.  If you have a personal problem, you can seek help from him.  Your supervisor recognizes when something work-related is going wrong.  Your supervisor gives you the freedom to handle difficult situations in the way that you feel is best.  He would not compromise ethical principles in order to achieve success. |

*Note.* The German original scenarios are adapted to be equal in length, cues for leader gender, and cues addressing the reader (e.g., “Ihnen”) but this does not account for the English translation.

**Supplementary Table 5.** Items of instrumental manipulation check for the written scenario.

|  | **German Original** | **English Translation** |
| --- | --- | --- |
| **Gender** | **Welches Geschlecht hatte die Person, die beschrieben wurde?**  Weiblich  Männlich  Kein Geschlecht genannt  Ich weiß nicht | **What is the gender of the described person?**  Female  Male  No gender mentioned  I do not know |
| **Position in Organization** | **Welche Person wurde im schriftlichen Szenario beschrieben?**  Mein/e Vorgesetzte/r  Mein/e Kollege/in  CEO meiner Firma | **Which person was described in the written scenario?**  My supervisor  My colleague  CEO of my company |
| **General Leadership Behavior** | **Welches Verhalten zeigte die beschriebene Person?**  …stellt den Leistungsanspruch und die Erwartungen an die Mitarbeitenden an oberste Stelle, indem die Person vorschreibt, wie Aufgaben ausgeführt werden sollen.  … stellt die Karriereentwicklung und das Wohl der Mitarbeitenden an oberste Stelle, indem die Person ermöglicht, sich in einem vertrauensvollen Umfeld zu entfalten.  Ich weiß es nicht. | **What behavior did the described person exhibit?**  ...puts the performance requirement and the expectations on the employees first place by dictating how tasks should be carried out.  ...puts career development and the well-being of employees first place by allowing the person to flourish in a trusted environment.  I do not know. |

- 1. **Supplementary Results**

All correlations of dependent variables, mediators, moderator, covariates, and demographics are depicted in Supplementary Table 6.

Regarding H1 and H2, the dependent variable perceived leader prototypicality deviated from linearity as assessed by scatterplots. We found homogeneity of the error variances for all dependent variables except prototypicality (*p* > .05). There was no homogeneity of covariances, as assessed by Box’s test (*p* > .001). These violations of the prerequisites for the MANOVA however decrease the statistical power of our analyses. Hence, our tests can be considered stricter.

Regarding H3 to H4, the results of the serial moderated mediation analysis are depicted in Supplementary Table 7 to Supplementary Table 14.

As an additional finding and to assess possible implicit age stereotypes evoked by the scenario manipulation, we asked participants to indicate the age of the respective leader and computed unpaired t-tests to compare the average ratings.^^[[2]](#footnote-2)^^ On average, raters perceived the servant leader (*M* = 40.82, *SD* = 7.19) to be significantly younger than the directive leaders (*M* = 43.39, *SD* = 7.75, *t*(450.91) = 3.66, *p* < .001, 95% BCa CI [1.19, 3.94]). Women leaders (*M* = 40.66, *SD* = 7.10) were perceived to be significantly younger than men leaders (*M* = 43.93, *SD* = 7.78, *t*(414.28) = 4.62, *p* < .001, 95% BCa CI [1.94, 4.57]).

**Supplementary Table 6.** Correlations of dependent variables, mediators, moderator, covariates, and demographics.

|  |  | 1 | 2 | 3 | 4 | 5 | 6 | 7 | 8 | 9 | 10 | 11 | 12 | 13 | 14 | 15 | 16 | 17 |
| --- | --- | --- | --- | --- | --- | --- | --- | --- | --- | --- | --- | --- | --- | --- | --- | --- | --- | --- |
| 1 | Effectiveness |  |  |  |  |  |  |  |  |  |  |  |  |  |  |  |  |  |
| 2 | Promotability | .86^**^ |  |  |  |  |  |  |  |  |  |  |  |  |  |  |  |  |
| 3 | Leader Liking | .84^**^ | .85^**^ |  |  |  |  |  |  |  |  |  |  |  |  |  |  |  |
| 4 | Prototypicality | .14^**^ | -.01 | -.06 |  |  |  |  |  |  |  |  |  |  |  |  |  |  |
| 5 | Warmth | .66^**^ | .68^**^ | .78^**^ | -.23^**^ |  |  |  |  |  |  |  |  |  |  |  |  |  |
| 6 | Morality | .75^**^ | .74^**^ | .79^**^ | -.06 | .85^**^ |  |  |  |  |  |  |  |  |  |  |  |  |
| 7 | Competence | .73^**^ | .68^**^ | .65^**^ | .18^**^ | .61^**^ | .80^**^ |  |  |  |  |  |  |  |  |  |  |  |
| 8 | Assertiveness | .53^**^ | .45^**^ | .40^**^ | .28^**^ | .34^**^ | .55^**^ | .79^**^ |  |  |  |  |  |  |  |  |  |  |
| 9 | Dominance | -.70^**^ | -.74^**^ | -.81^**^ | .23^**^ | -.82^**^ | -.75^**^ | -.50^**^ | -.24^**^ |  |  |  |  |  |  |  |  |  |
| 10 | Rater EGRB  **Covariates** | .06 | .06 | .04 | -.08 | .02 | .10^*^ | .13^**^ | .13^**^ | -.07 |  |  |  |  |  |  |  |  |
| 11 | Rater gender | .10^*^ | .10^*^ | .09^*^ | .00 | .04 | .08 | .09 | .05 | -.07 | .30^**^ |  |  |  |  |  |  |  |
| 12 | Rater age | -.10^*^ | -.12^*^ | -.08 | -.04 | -.08 | -.11^*^ | -.10^*^ | -.06 | .06 | .04 | -.22^**^ |  |  |  |  |  |  |
| 13 | Rater’s managerial responsibility  **Demographics** | -.12^**^ | -.09 | -.04 | -.03 | -.04 | -.06 | -.12^**^ | -.04 | .05 | -.20^**^ | -.11^*^ | .06 |  |  |  |  |  |
| 14 | Work time | -.07 | -.05 | -.03 | -.03 | .01 | -.01 | -.06 | -.05 | -.01 | -.10^*^ | -.18^**^ | .07 | .17^**^ |  |  |  |  |
| 15 | Education | .10^*^ | .14^**^ | .13^**^ | -.03 | .11^*^ | .13^**^ | .11^*^ | .02 | -.13^**^ | -.04 | -.02 | .13^**^ | -.04 | .00 |  |  |  |
| 16 | Having (had) a supervisor | .07 | .08 | .08 | .02 | .01 | .03 | .03 | .01 | -.04 | .04 | .02 | -.02 | -.10^*^ | .03 | .00 |  |  |
| 17 | Thinking of supervisor | .08 | .08 | .11^*^ | .08 | -.01 | .01 | .06 | .06 | -.03 | .00 | .07 | -.06 | .01 | -.06 | .01 | .05 |  |
| 18 | Imagination of scenario | -.25^**^ | -.18^**^ | -.20^**^ | -.31^**^ | -.08 | -.21^**^ | -.34^**^ | -.35^**^ | .06 | -.10^*^ | -.05 | .08 | .01 | -.05 | -.02 | .03 | -.12^*^ |

*Note.* *N* = 454. Age in years. Men are coded as 0, women are coded as 1. Rater’s managerial responsibility, no coded as 0, yes coded as 1. Having had a supervisor, no coded as 0, yes coded as 1. Thinking of supervisor, no coded as 0, yes coded as 1. Imagination of scenario ranging from 1 to 5 with higher scores indicating worse imagination. EGRB, Egalitarian gender role beliefs. **p* < .05; ***p* < .01.

**Supplementary Table 7.** Results of the serial moderated mediation analysis for Hypothesis 3a).

| **Outcome** | *B* | *SE (HC3)* | *t* | *95% CI* |
| --- | --- | --- | --- | --- |
| **Perceived Warmth** |  |  |  |  |
| **Predictors** |  |  |  |  |
| Leadership behavior | 1.49** | 0.09 | 15.99** | (1.31; 1.66) |
| Leader gender | -0.08 | 0.09 | -0.80 | (-0.26; 0.11) |
| Leadership behavior*Leader gender | -0.07 | 0.13 | -0.55 | (-0.33; 0.18) |
| Rater age | -0.06 | 0.03 | -1.66 | (-0.13; 0.01) |
| Rater gender | 0.01 | 0.07 | 0.08 | (-0.13; 0.14) |
| Rater’s managerial responsibility | -0.10 | 0.08 | -1.28 | (-0.26; 0.05) |
|  |  |  |  |  |
| **Perceived Leader Effectiveness** | |  |  |  |
| **Predictors** |  |  |  |  |
| Leadership behavior | 0.17 | 0.13 | 1.33 | (-0.08; 0.42) |
| Perceived Warmth | 0.59** | 0.07 | 8.47** | (0.46; 0.72) |
| Rater age | -0.04 | 0.04 | -1.02 | (-0.11; 0.03) |
| Rater gender | 0.12 | 0.07 | 1.57 | (-0.03; 0.26) |
| Rater’s managerial responsibility | -0.20* | 0.08 | -2.39* | (-0.37; -0.03) |
|  |  |  |  |  |
| **Perceived Leader Promotability** | |  |  |  |
| **Predictors** |  |  |  |  |
| Leadership behavior | 0.24** | 0.07 | 3.45** | (0.10; 0.37) |
| Perceived Warmth | 0.12* | 0.05 | 2.49* | (0.03; 0.21) |
| Perceived Leader Effectiveness | 0.71** | 0.04 | 18.47** | (0.64; 0.79) |
| Rater age | -0.03 | 0.03 | -1.14 | (-0.08; 0.02) |
| Rater gender | 0.01 | 0.05 | 0.23 | (-0.08; 0.10) |
| Rater’s managerial responsibility | 0.02 | 0.05 | 0.30 | (-0.09; 0.12) |

*Note.* **p* < .05; ***p* < .01. Directive leadership is coded as 0, servant leadership as 1. Men are coded as 0, women are coded as 1. Rater’s managerial responsibility, no coded as 0, yes coded as 1.

**Supplementary Table 8.** Results of the serial moderated mediation analysis for Hypothesis 3a).

| **Outcome** | *B* | *SE (HC3)* | *t* | *95% CI* |
| --- | --- | --- | --- | --- |
| **Perceived Warmth** |  |  |  |  |
| **Predictors** |  |  |  |  |
| Leadership behavior | 1.49** | 0.09 | 15.99** | (1.31; 1.66) |
| Leader gender | -0.08 | 0.09 | -0.80 | (-0.26; 0.11) |
| Leadership behavior*Leader gender | -0.07 | 0.13 | -0.55 | (-0.33; 0.19) |
| Rater age | -0.06 | 0.03 | -1.66 | (-0.13; 0.01) |
| Rater gender | 0.01 | 0.07 | 0.08 | (-0.13; 0.14) |
| Rater’s managerial responsibility | -0.10 | 0.08 | -1.28 | (-0.26; 0.05) |
|  |  |  |  |  |
| **Perceived Leader Liking** |  |  |  |  |
| **Predictors** |  |  |  |  |
| Leadership behavior | 0.34** | 0.13 | 2.70** | (0.08; 0.58) |
| Perceived Warmth | 0.65** | 0.06 | 9.96** | (0.53; 0.79) |
| Rater age | -0.01 | 0.03 | -0.39 | (-0.07; 0.05) |
| Rater gender | 0.11 | 0.06 | 1.83 | (-0.01; 0.23) |
| Rater’s managerial responsibility | -0.01 | 0.07 | -0.21 | (-0.15; 0.11) |
|  |  |  |  |  |
| **Perceived Leader Promotability** | | |  |  |
| **Predictors** |  |  |  |  |
| Leadership behavior | 0.08 | 0.06 | 1.32 | (-0.04; 0.21) |
| Perceived Warmth | 0.01 | 0.05 | 0.13 | (-0.08; 0.11) |
| Perceived Leader Liking | 0.81** | 0.05 | 17.49** | (0.72; 0.90) |
| Rater age | -0.05 | 0.03 | -1.80 | (-0.10; 0.00) |
| Rater gender | 0.00 | 0.05 | 0.04 | (-0.10; 0.10) |
| Rater’s managerial responsibility | -0.12* | 0.06 | -2.04* | (-0.23; -0.01) |

*Note.* **p* < .05; ***p* < .01. Directive leadership is coded as 0, servant leadership as 1. Men are coded as 0, women are coded as 1. Rater’s managerial responsibility, no coded as 0, yes coded as 1.

**Supplementary Table 9**. Results of the serial moderated mediation analysis for Hypothesis 3b).

| **Outcome** | *B* | *SE (HC3)* | *t* | *95% CI* |
| --- | --- | --- | --- | --- |
| **Perceived Morality** |  |  |  |  |
| **Predictors** |  |  |  |  |
| Leadership behavior | 1.20** | 0.11 | 10.70** | (0.98; 1.41) |
| Leader gender | -0.03 | 0.11 | -0.28 | (-0.25; 0.19) |
| Leadership behavior*Leader gender | -0.14 | 0.16 | -0.87 | (-0.44; 0.17) |
| Rater age | -0.09 | 0.04 | -1.96 | (-0.17; 0.00) |
| Rater gender | 0.08 | 0.09 | 0.89 | (-0.09; 0.24) |
| Rater’s managerial responsibility | -0.13 | 0.09 | -1.44 | (-0.31; 0.04) |
|  |  |  |  |  |
| **Perceived Leader Effectiveness** | |  |  |  |
| **Predictors** |  |  |  |  |
| Leadership behavior | 0.29** | 0.10 | 2.79** | (0.09; 0.48) |
| Perceived Morality | 0.66** | 0.06 | 10.28** | (0.54; 0.78) |
| Rater age | -0.02 | 0.03 | -0.52 | (-0.07; 0.04) |
| Rater gender | 0.07 | 0.07 | 1.01 | (-0.06; 0.19) |
| Rater’s managerial responsibility | -0.18* | 0.08 | -2.23* | (-0.33; -0.02) |
|  |  |  |  |  |
| **Perceived Leader Promotability** | |  |  |  |
| **Predictors** |  |  |  |  |
| Leadership behavior | 0.29** | 0.06 | 4.68** | (0.17; 0.40) |
| Perceived Morality | 0.15** | 0.04 | 3.57** | (0.07; 0.24) |
| Perceived Leader Effectiveness | 0.66** | 0.04 | 16.57** | (0.58; 0.74) |
| Rater age | -0.03 | 0.03 | -1.07 | (-0.08; 0.02) |
| Rater gender | 0.01 | 0.05 | 0.12 | (-0.08; 0.10) |
| Rater’s managerial responsibility | 0.01 | 0.05 | 0.21 | (-0.09; 0.12) |

*Note.* **p* < .05; ***p* < .01. Directive leadership is coded as 0, servant leadership as 1. Men are coded as 0, women are coded as 1. Rater’s managerial responsibility, no coded as 0, yes coded as 1.

**Supplementary Table 10**. Results of the serial moderated mediation analysis for Hypothesis 3b).

| **Outcome** | *B* | *SE (HC3)* | *t* | 95% CI |
| --- | --- | --- | --- | --- |
| **Perceived Morality** |  |  |  |  |
| **Predictors** |  |  |  |  |
| Leadership behavior | 1.20** | 0.11 | 10.70** | (0.98; 1.41) |
| Leader gender | -0.03 | 0.11 | -0.28 | (-0.25; 0.19) |
| Leadership behavior*Leader gender | -0.14 | 0.16 | -0.87 | (-0.44; 0.17) |
| Rater age | -0.09 | 0.04 | -1.96 | (-0.17; 0.00) |
| Rater gender | 0.08 | 0.09 | 0.89 | (-0.09; 0.24) |
| Rater’s managerial responsibility | -0.13 | 0.09 | -1.44 | (-0.31; 0.04) |
|  |  |  |  |  |
| **Perceived Leader Liking** |  |  |  |  |
| **Predictors** |  |  |  |  |
| Leadership behavior | 0.59** | 0.09 | 6.34** | (0.41; 0.76) |
| Perceived Morality | 0.62** | 0.05 | 11.30** | (0.52; 0.73) |
| Rater age | 0.00 | 0.03 | 0.10 | (-0.05; 0.05) |
| Rater gender | 0.07 | 0.06 | 1.16 | (-0.05; 0.18) |
| Rater’s managerial responsibility | 0.00 | 0.06 | 0.05 | (-0.12; 0.13) |
|  |  |  |  |  |
| **Perceived Leader Promotability** | |  |  |  |
| **Predictors** |  |  |  |  |
| Leadership behavior | 0.06 | 0.06 | 0.99 | (-0.06; 0.18) |
| Perceived Morality | 0.15* | 0.05 | 2.99* | (0.06; 0.25) |
| Perceived Leader Liking | 0.71** | 0.05 | 13.62** | (0.60; 0.81) |
| Rater age | -0.04 | 0.02 | -1.56 | (-0.09; 0.01) |
| Rater gender | 0.00 | 0.05 | 0.06 | (-0.10; 0.10) |
| Rater’s managerial responsibility | -0.11 | 0.06 | -1.89 | (-0.22; 0.00) |

*Note.* **p* < .05; ***p* < .01. Directive leadership is coded as 0, servant leadership as 1. Men are coded as 0, women are coded as 1. Rater’s managerial responsibility, no coded as 0, yes coded as 1.

**Supplementary Table 11**. Results of the serial moderated mediation analysis for Hypothesis 4a).

| **Outcome** | *B* | *SE (HC3)* | *t* | 95% CI |
| --- | --- | --- | --- | --- |
| **Perceived Competence** |  |  |  |  |
| **Predictors** |  |  |  |  |
| Leadership behavior | 0.62** | 0.13 | 4.60** | (0.36; 0.89) |
| Leader gender | -0.11 | 0.13 | -0.81 | (-0.37; 0.15) |
| Leadership behavior*Leader gender | 0.02 | 0.18 | 0.09 | (-0.33; 0.37) |
| Rater age | -0.08 | 0.05 | -1.61 | (-0.17; 0.01) |
| Rater gender | 0.09 | 0.09 | 1.00 | (-0.10; 0.28) |
| Rater’s managerial responsibility | -0.26* | 0.10 | -2.51* | (-0.47; -0.06) |
|  |  |  |  |  |
| **Perceived Leader Effectiveness** | |  |  |  |
| **Predictors** |  |  |  |  |
| Leadership behavior | 0.64** | 0.08 | 9.11** | (0.50; 0.78) |
| Perceived Competence | 0.62** | 0.05 | 12.33** | (0.52; 0.72) |
| Rater age | -0.02 | 0.03 | -0.79 | (-0.08; 0.03) |
| Rater gender | 0.06 | 0.06 | 0.99 | (-0.06; 0.18) |
| Rater’s managerial responsibility | -0.10 | 0.07 | -1.35 | (-0.24; 0.04) |
|  |  |  |  |  |
| **Perceived Leader Promotability** | |  |  |  |
| **Predictors** |  |  |  |  |
| Leadership behavior | 0.38** | 0.06 | 6.57** | (0.27; 0.49) |
| Perceived Competence | 0.13** | 0.04 | 3.25** | (0.05; 0.21) |
| Perceived Leader Effectiveness | 0.66** | 0.04 | 16.57** | (0.58; 0.74) |
| Rater age | -0.03 | 0.03 | -1.16 | (-0.08; 0.02) |
| Rater gender | 0.01 | 0.05 | 0.13 | (-0.09; 0.10) |
| Rater’s managerial responsibility | 0.02 | 0.05 | 0.46 | (-0.08; 0.13) |

*Note.* **p* < .05; ***p* < .01. Directive leadership is coded as 0, servant leadership as 1. Men are coded as 0, women are coded as 1. Rater’s managerial responsibility, no coded as 0, yes coded as 1.

**Supplementary Table 12.** Results of the serial moderated mediation analysis for Hypothesis 4a).

| **Outcome** | *B* | *SE (HC3)* | *t* | *95% CI* |
| --- | --- | --- | --- | --- |
| **Perceived Competence** |  |  |  |  |
|  |  |  |  |  |
| **Predictors** |  |  |  |  |
| Leadership behavior | 0.62** | 0.13 | 4.60** | (0.36; 0.89) |
| Leader gender | -0.11 | 0.13 | -0.81 | (-0.37; 0.15) |
| Leadership behavior*Leader gender | 0.02 | 0.18 | 0.09 | (-0.33; 0.37) |
| Rater age | -0.08 | 0.05 | -1.61 | (-0.17; 0.01) |
| Rater gender | 0.09 | 0.09 | 1.00 | (-0.10; 0.28) |
| Rater’s managerial responsibility | -0.26* | 0.10 | -2.51* | (-0.47; -0.06) |
|  |  |  |  |  |
| **Perceived Leader Liking** |  |  |  |  |
| **Predictors** |  |  |  |  |
| Leadership behavior | 0.98** | 0.07 | 14.19** | (0.85; 1.11) |
| Perceived Competence | 0.49** | 0.04 | 11.37** | (0.41; 0.57) |
| Rater age | -0.01 | 0.03 | -0.38 | (-0.07; 0.05) |
| Rater gender | 0.07 | 0.06 | 1.18 | (-0.04; 0.19) |
| Rater’s managerial responsibility | 0.05 | 0.07 | 0.75 | (-0.09; 0.18) |
|  |  |  |  |  |
| **Perceived Leader Promotability** | |  |  |  |
| **Predictors** |  |  |  |  |
| Leadership behavior | 0.16** | 0.06 | 2.65** | (0.04; 0.28) |
| Perceived Competence | 0.22** | 0.04 | 4.95** | (0.14; 0.31) |
| Perceived Leader Liking | 0.65** | 0.05 | 14.01** | (0.56; 0.74) |
| Rater age | -0.04 | 0.03 | -1.52 | (-0.09; 0.01) |
| Rater gender | 0.00 | 0.05 | 0.02 | (-0.09; 0.09) |
| Rater’s managerial responsibility | -0.07 | 0.05 | -1.35 | (-0.18; 0.03) |

*Note.* **p* < .05; ***p* < .01. Directive leadership is coded as 0, servant leadership as 1. Men are coded as 0, women are coded as 1. Rater’s managerial responsibility, no coded as 0, yes coded as 1.

**Supplementary Table 13.** Results of the serial moderated mediation analysis for Hypothesis 4b).

| **Outcome** | *B* | *SE (HC3)* | *t* | *95% CI* |
| --- | --- | --- | --- | --- |
| **Perceived Dominance** |  |  |  |  |
| **Predictors** |  |  |  |  |
| Leadership behavior | -1.47** | 0.09 | 15.75** | (-1.66; -1.29) |
| Leader gender | 0.07 | 0.10 | 0.75 | (-0.11; 0.26) |
| Leadership behavior*Leader gender | 0.01 | 0.13 | 0.10 | (-0.23; 0.26) |
| Rater age | 0.03 | 0.03 | 0.90 | (-0.04; 0.09) |
| Rater gender | -0.07 | 0.07 | -1.04 | (-0.20; 0.06) |
| Rater’s managerial responsibility | 0.10 | 0.08 | 1.33 | (-0.04; 0.25) |
|  |  |  |  |  |
| **Perceived Leader Effectiveness** | |  |  |  |
| **Predictors** |  |  |  |  |
| Leadership behavior | 0.06 | 0.10 | 0.52 | (-0.16; 0.26) |
| Perceived Dominance | -0.66** | 0.06 | -11.60** | (-0.78; -0.55) |
| Rater age | -0.05 | 0.03 | -1.42 | (-0.12; 0.02) |
| Rater gender | 0.07 | 0.07 | 1.05 | (-0.06; 0.20) |
| Rater’s managerial responsibility | -0.19* | 0.08 | -2.28* | (-0.36; -0.03) |
|  |  |  |  |  |
| **Perceived Leader Promotability** | |  |  |  |
| **Predictors** |  |  |  |  |
| Leadership behavior | 0.12 | 0.06 | 1.84 | (-0.01; 0.24) |
| Perceived Dominance | -0.24** | 0.04 | -5.24** | (-0.32; -0.15) |
| Perceived Leader Effectiveness | 0.66** | 0.04 | 16.57** | (0.58; 0.74) |
| Rater age | -0.03 | 0.02 | -1.34 | (-0.08; 0.01) |
| Rater gender | 0.00 | 0.05 | 0.05 | (-0.09; 0.09) |
| Rater’s managerial responsibility | 0.01 | 0.05 | 0.26 | (-0.09; 0.11) |

*Note.* **p* < .05; ***p* < .01. Directive leadership is coded as 0, servant leadership as 1. Men are coded as 0, women are coded as 1. Rater’s managerial responsibility, no coded as 0, yes coded as 1.

**Supplementary Table 14.** Results of the serial moderated mediation analysis for Hypothesis 4b).

| **Outcome** | *B* | *SE (HC3)* | *t* | *95% CI* |
| --- | --- | --- | --- | --- |
| **Perceived Dominance** |  |  |  |  |
| **Predictors** |  |  |  |  |
| Leadership behavior | -1.47** | 0.09 | 15.75** | (-1.66; -1.29) |
| Leader gender | 0.07 | 0.10 | 0.75 | (-0.11; 0.26) |
| Leadership behavior*Leader gender | 0.01 | 0.13 | 0.10 | (-0.23; 0.26) |
| Rater age | 0.03 | 0.03 | 0.90 | (-0.04; 0.09) |
| Rater gender | -0.07 | 0.07 | -1.04 | (-0.20; 0.06) |
| Rater’s managerial responsibility | 0.10 | 0.08 | 1.33 | (-0.04; 0.25) |
|  |  |  |  |  |
| **Perceived Leader Liking** |  |  |  |  |
| **Predictors** |  |  |  |  |
| Leadership behavior | 0.23* | 0.09 | 2.51* | (0.05; 0.40) |
| Perceived Dominance | -0.72** | 0.05 | -15.36** | (-0.81; -0.63) |
| Rater age | -0.03 | 0.03 | -0.94 | (-0.08; 0.03) |
| Rater gender | 0.07 | 0.06 | 1.17 | (-0.04; 0.18) |
| Rater’s managerial responsibility | -0.01 | 0.07 | -0.08 | (-0.14; 0.13) |
|  |  |  |  |  |
| **Perceived Leader Promotability** | |  |  |  |
| **Predictors** |  |  |  |  |
| Leadership behavior | -0.01 | 0.07 | -0.22 | (-0.14; 0.12) |
| Perceived Dominance | -0.14** | 0.05 | -2.75** | (-0.24; -0.04) |
| Perceived Leader Liking | 0.74** | 0.05 | 14.87** | (0.64; 0.83) |
| Rater age | -0.05 | 0.03 | -1.83 | (-0.09; 0.00) |
| Rater gender | 0.00 | 0.05 | 0.03 | (-0.10; 0.10) |
| Rater’s managerial responsibility | -0.11 | 0.06 | -1.94 | (-0.22; 0.00) |

*Note.* **p* < .05; ***p* < .01. Directive leadership is coded as 0, servant leadership as 1. Men are coded as 0, women are coded as 1. Rater’s managerial responsibility, no coded as 0, yes coded as 1.

**3.2.1 Assertiveness**

*Assertiveness.* For completeness of Abele et al.’s scale (2016), we also assessed perceived leader assertiveness. Assertiveness is related to perceived leader promotability (i.e., ambitious agency; Ma et al., 2022) and has an inverted u-shaped relationship with perceived leader effectiveness, with unfavorable evaluations of leaders who display low or high assertiveness (Ames, 2009; Ames & Flynn, 2007). As assertiveness seems to be a leadership weakness instead of a strength (Ames & Flynn, 2007), we did not expect it to explain leadership outcomes.

*Confirmatory Factor Analysis.* Besides our hypothesized 10-factor model, we computed a confirmatory factor analysis in R with *lavaan* to examine the fit with the data, including the third subdimension of agency, assertiveness, that we assessed for exploratory purposes. The results for this proposed 10-factor model suggests a good fit with the data, χ2(2034, 454) = 4804.946, *p* < .001, CFI = 0.907, TLI = 0.901, RMSEA = .055, SRMR = .070, compared to a single factor model, χ2(2079, 454) = 15404.354, *p* < .001, CFI = 0.551, TLI = 0.536, RMSEA = 0.119, SRMR = 0.151, Δχ2(45) = 10599, *p* < .001. An eight-factor model merging warmth and morality into the factor communion and merging competence and assertiveness into the factor agency, χ2(2051, 454) = 5255.510, *p* < .001, CFI = 0.892, TLI = 0.887, RMSEA = .059, SRMR = .075, Δχ2(17) = 450.56, *p* < .001, as well as a seven-factor model including dominance into the factor agency, χ2(2058, 454) = 6752.789, *p* < .001, CFI = 0.842, TLI = 0.835, RMSEA = .071, SRMR = .094, Δχ2(24) = 1947.80, *p* < .001, showed a worse fit with the data.

*Exploratory Analysis.* We computed an ANCOVA as an exploratory analysis with SPSS 27 to examine the main effects of leadership behavior and leader gender and their interaction on perceived leader assertiveness. We included rater gender, rater age, and rater managerial responsibility as covariates. We found no significant main effect of leadership behavior (*F*(1, 447) = 1.77, *p* = 0.19, partial *η*² = 0.00) and no interaction effect of leadership behavior and leader gender (*F*(1, 447) = 0.07, *p* = 0.79, partial *η*² = 0.00) on perceived assertiveness. Interestingly, we found a significant main effect of leader gender on perceived leader assertiveness (*F*(1, 447) = 5.35, *p* = 0.02, partial *η*² = 0.01). Pairwise comparisons with bootstrapping (1,000 resamples) revealed that women were perceived as less assertive than men (*M*_W_ = 3.60, *M*_M_ = 3.77, *p* = 0.03, 95% CI [-0.34, -0.04]). These results are consistent with findings that women are rated as less assertive than men (Hentschel et al., 2019).

We also computed a moderated mediation analysis with PROCESS macro, version 4.2, Hayes (2018, Model 83) in SPSS 27 to compute moderated mediation analyses using bootstrapping (5,000 resamples) with 95% bias-corrected confidence intervals of the indirect effects and z-standardized scales. We included the same covariates. As independent variable, we included leadership behavior. The moderator variable was leader gender. The first stage mediator was leader assertiveness, and the second stage mediator was either perceived leader effectiveness or liking. The confidence intervals of the indirect mediation effect included zero for women (via effectiveness:  *B* = 0.05, *SE* = 0.05, 95 % CI [-0.03, 0.15], via liking: *B* = 0.04, *SE* = 0.03, 95 % CI [-0.02, 0.11]) and for men leaders (via effectiveness: *B* = 0.04, *SE* = 0.05, 95 % CI [-0.06, 0.14], via liking: *B* = 0.03, *SE* = 0.04, 95 % CI [-0.05, 0.10]). The confidence intervals of the indirect mediation effect and the index of the moderated mediation effect included zero (see Supplementary Table 15). Thus, leader assertiveness did not mediate the relationship between leadership behavior and leader promotability via leader effectiveness and leader liking, neither for women leaders nor men leaders.

**Supplementary Table 15.** Exploratory indirect effects and index of the moderated serial mediation for leader assertiveness.

| **Condition** | **Predictor** | **1^st^ Mediator** | **2^nd^ Mediator** | **Outcome** | **Path** | ***B*** | ***SE*** | ***95% CI*** |
| --- | --- | --- | --- | --- | --- | --- | --- | --- |
| Woman | Leadership behavior | Assertiveness | Effectiveness | Promotability | X M1 M2 O | 0.05 | 0.05 | [-0.03, 0.15] |
| Man | Leadership behavior | Assertiveness | Effectiveness | Promotability | X M1 M2 O | 0.04 | 0.05 | [-0.06, 0.14] |
| **Index of moderated mediation** | | | | | | 0.02 | 0.07 | [-0.11, 0.16] |
| Woman | Leadership behavior | Assertiveness | Liking | Promotability | X M1 M2 O | 0.04 | 0.03 | [-0.02, 0.11] |
| Man | Leadership behavior | Assertiveness | Liking | Promotability | X M1 M2 O | 0.03 | 0.04 | [-0.05, 0.10] |
| **Index of moderated mediation** | | |  |  |  | 0.01 | 0.05 | [-0.08, 0.12] |

*Note.* *N* = 454. The moderated mediations included the covariates rater age, rater gender, and raters’ managerial responsibility. The indirect effects and the index of the moderated mediation were computed using bootstrapping (5,000 resamples).

**3.2.2 Raters’ Gender Role Beliefs**

*Gender Role Beliefs.* Depending on socialization and daily observation (Eagly & Karau, 2002; Eagly & Wood, 2012), people may hold more egalitarian or traditional gender role beliefs. Raters with egalitarian gender role beliefs consider women and men equally capable in decision-making and caretaking roles. Raters with traditional gender role beliefs believe in a gender-biased social role division as portrayed in images of women as caretakers and men as decision-makers (Eagly & Wood, 2012; Koenig & Eagly, 2014). They justify the current gender hierarchy (Kray et al., 2017; see *system justification theory*; Jost et al., 2004) and give more gender-biased evaluations evident in lower hireability and liking of agentic women compared to agentic men (Rudman et al., 2012). In line with this, role congruity theory posits that raters’ gender role beliefs influence evaluations of women leaders vs. men leaders (Eagly & Karau, 2002).

*Egalitarian Gender Role Beliefs, Leadership Behavior, and Leader Gender.* We expect that the proposed effects of servant vs. directive leadership on leader promotability and prototypicality differ for raters with egalitarian vs. traditional gender role beliefs. We argue that raters with rather egalitarian gender role beliefs will likely support a violation of the gender hierarchy. According to their egalitarian beliefs, such a gender hierarchy should not exist. Thus, their evaluation of stereotype-violating leaders is likely even more positive than that of stereotype-conforming leaders. Regarding leader prototypicality, egalitarian raters likely perceive a lower incongruence between the gender role and the leadership behavior of stereotype-violating leaders. This lower incongruence should result in a more positive evaluation of leader prototypicality for stereotype-violating leaders than stereotype-conforming leaders.

*Traditional Gender Role Beliefs, Leadership Behavior, and Leader Gender.* We argue that raters with traditional gender role beliefs will perceive any violation of the gender hierarchy as negative. They likely perceive servant men leaders to violate men’s high status because servant men leaders demonstrate high communion (Moss-Racusin et al., 2010; Rudman et al., 2012). The same holds for directive women leaders who are likely perceived to violate women’s low status by showing high agency (Rudman et al., 2012). Traditional raters likely seek to restore women’s lower and men’s higher positions in the gender system (Rudman et al., 2012). Thus, they should penalize gender role incongruent leadership behavior (Jussim et al., 1987; Prentice & Carranza, 2004; Eagly & Karau, 2002). Hence, when raters hold traditional gender role beliefs, we propose an evaluative penalty for stereotype-violating leaders compared to stereotype-conforming leaders. Regarding leader prototypicality, raters with traditional gender role beliefs likely perceive stereotype-violating leaders as even less prototypical due to an even higher perceived incongruence between the gender role and the leadership behavior. Thus, we propose:

*Hypothesis 5: Raters’ gender role beliefs moderate the proposed (mediated) relationship such that when beliefs are egalitarian (compared to traditional), the evaluation of perceived leader promotability (H5a) and leader prototypicality (H5b) is more positive for directive women leaders compared to directive men leaders and for servant men leaders compared to servant women leaders.*

*Gender role beliefs* were assessed by an adapted version of Larsen and Long’s (1988) 20-item comprising *Attitudes toward Sex Roles Scale* (α = 0.92). We exchanged the term “wife” and “husband” with “woman” and “man”. The scale includes eight statements about egalitarian beliefs (e.g., “Having a job is just as important for a woman as it is for a man.”) and 12 statements regarding traditional beliefs (e.g., “Men make better leaders.”). As we reversed the 12 traditional statements and averaged all items in line with Larsen and Long (1988). A preference for egalitarian gender roles is represented by higher values (1 = *do not agree at all* to 7 = *totally agree*). The data were strongly skewed towards egalitarian gender role beliefs (*M* = 5.93, *SD* = 0.92, *Min* = 3, *Max* = 7).

*Results.* To test H5a, we tested the full moderated serial mediation analysis in MPLUS Version 8.7 (Muthén & Muthén, 1998–2017). We tested the hypothesis using bootstrapping (10,000 resamples) with 95% bias-corrected confidence intervals of the hypothesized indirect effects. Because one moderator, traditional gender role beliefs, was strongly skewed towards egalitarian gender role beliefs, we median-centered this variable to receive more interpretable estimates (Field, 2018). In our study, a low level of egalitarian gender role beliefs thus represents a high level of egalitarian gender role beliefs in reality, a medium level represents an actually very high level, and a high level represents an extremely high level of egalitarian gender role beliefs. We again used the same covariates as before. We included leadership behavior as independent variable. The first moderator variable of the moderated moderation was leader gender, and the second moderator was raters’ gender role beliefs. At the first mediator stage, we simultaneously included perceived warmth, morality, competence, and dominance; at the second mediator stage, we simultaneously included perceived leader effectiveness and liking. We included perceived leader promotability as dependent variable. Before analysis, we z-standardized all continuous variables. We found no moderated effect, as the tests of highest-order unconditional interactions were non-significant. The confidence interval of the interaction effect on warmth (*B* *=* -0.00, *SE* = 0.08, 95% CI [-0.16; 0.16]), morality (*B* *=* 0.01, *SE* = 0.09, 95% CI [-0.17; 0.20]), competence (*B* *=* -0.01, *SE* = 0.11, 95% CI [-0.22; 0.20]), and dominance (*B* *=* 0.01, *SE* = 0.08, 95% CI [-0.16; 0.17]) included zero (see Supplementary Table 16 for the output of the moderated mediation model). As almost all participants were at least moderately egalitarian, this does not allow us to definitely conclude that H5a was not supported.

To test H5b, we used the PROCESS macro, version 4.2, Hayes (2018) in SPSS 27 to compute the moderated moderation analyses. We tested the hypothesis using bootstrapping (5,000 resamples) with 95% bias-corrected confidence intervals of the hypothesized indirect effects. The same covariates were used. We implemented Model 3. As independent variable we included leadership behavior. The first moderator variable of the moderated moderation was leader gender, and the second moderator was raters’ gender role beliefs.^^[[3]](#footnote-3)^^ As a dependent variable, we included perceived leader prototypicality. Prior to analysis, we z-standardized all continuous variables. Leadership behavior significantly predicted leader prototypicality (*B* *=* -0.86, *SE* = 0.13, 95% CI [-1.12, -0.60]). Yet, there was no indication of a moderated effect, as the test of highest-order unconditional interaction was not significant. The confidence interval of the interaction effect on perceived leader prototypicality (*B* *=* 0.17, *SE* = 0.18, 95% CI [-0.16, 0.53]) included zero. As almost all participants were at least moderately egalitarian, this does not allow us to definitely conclude that H5b was not supported.

For the smaller sample (*N* = 337), we conducted the analyses as well, and the results did not differ. Interestingly, for H5a in the smaller sample, the confidence interval of the interaction effect between leadership behavior, leader gender, and rater gender role beliefs on warmth (*B* *=* 0.15, *SE* = 0.07, *p* = 0.03, 95% CI [0.03, 0.31]) and on morality (*B* *=* 0.16, *SE* = 0.08, *p* = 0.06, 95% CI [0.01, 0.34]) excluded zero. But only for warmth, the interaction effect became significant. Yet, warmth was not significantly related to perceived leader effectiveness (*B* *=* -0.12, *SE* = 0.09, 95% CI [-0.29, 0.07]), neither to leader liking (*B* *=* 0.09, *SE* = 0.10, 95% CI [-0.10, 0.28]) which were both related to leader promotability (effectiveness: *B* *=* 0.39, *SE* = 0.06, 95% CI [0.28, 0.50], liking: *B* *=* 0.33, *SE* = 0.06, 95% CI [0.22, 0.45]).

*Conclusion and Limitations.* Regarding raters’ characteristics as boundary conditions, we can neither confirm nor reject the propositions of role congruity theory (Eagly & Karau, 2002) that raters’ gender role beliefs influence gender-biased leadership evaluations. As our sample consisted only of rather egalitarian raters, these raters might not have perceived any expectancy violation. Instead, they might have equally expected women and men to implement the respective leadership behavior. This could also explain why the evaluations of servant and directive leadership in H2 to H4 did not differ according to leader gender.

A potential explanation for the skewness toward egalitarian gender role beliefs is that raters’ gender role beliefs might have been confounded by demand effects (Khademi et al., 2021). Participants had to answer the manipulation checks before we assessed gender role beliefs. The manipulation checks included the gender of the leader depicted in the scenario which might have revealed our gender hypothesis to the raters. In addition, being created more than 30 years ago, the scale may also be outdated. A more recent measure could be, for example, the *Belief in Sexism Shift Scale* (Zehnter et al., 2021). Alternatively, raters’ social desirability may have confounded the data distribution.

**Supplementary Table 16.** Results of the serial moderated mediation analysis for Hypothesis 5a).

| **Outcome** | *B* | *SE* | *t* | *95% CI* |
| --- | --- | --- | --- | --- |
| **Perceived Warmth** |  |  |  |  |
| **Predictors** |  |  |  |  |
| Leadership behavior | 0.77** | 0.04 | 18.55** | (0.68; 0.85) |
| Leader gender | -0.04 | 0.05 | -0.72 | (-0.13; 0.06) |
| Gender Role Beliefs (median-centered) | -0.04 | 0.08 | -0.46 | (-0.19; 0.14) |
| Leadership behavior*Leader gender | -0.04 | 0.06 | -0.58 | (-0.15; 0.09) |
| Leadership behavior*Gender Role Beliefs | 0.11 | 0.08 | 1.31 | (-0.06; 0.27) |
| Gender Role Beliefs*Leader Gender | -0.02 | 0.08 | -0.23 | (-0.17; 0.14) |
| Leadership behavior*Leader Gender* Gender Role Beliefs | 0.00 | 0.08 | -0.00 | (-0.16; 0.16) |
| Rater age | -0.06 | 0.03 | -1.73 | (-0.13; 0.01) |
| Rater gender | -0.01 | 0.04 | -0.15 | (-0.07; 0.63) |
| Rater’s managerial responsibility | -0.04 | 0.04 | -1.09 | (-0.12; 0.03) |
|  |  |  |  |  |
| **Perceived Morality** |  |  |  |  |
| **Predictors** |  |  |  |  |
| Leadership behavior | 0.60** | 0.05 | 11.30** | (0.50; 0.70) |
| Leader gender | -0.02 | 0.06 | -0.30 | (-0.17; 0.10) |
| Gender Role Beliefs (median-centered) | 0.09 | 0.09 | 0.95 | (-0.09; 0.28) |
| Leadership behavior*Leader gender | -0.06 | 0.07 | -0.77 | (-0.20; 0.09) |
| Leadership behavior*Gender Role Beliefs | 0.02 | 0.09 | 0.18 | (-0.16; 0.20) |
| Gender Role Beliefs*Leader Gender | -0.01 | 0.09 | -0.07 | (-0.19; 0.18) |
| Leadership behavior*Leader Gender* Gender Role Beliefs | 0.01 | 0.09 | 0.14 | (-0.17; 0.20) |
| Rater age | -0.10* | 0.04 | -2.32* | (-0.18; -0.02) |
| Rater gender | 0.01 | 0.04 | 0.19 | (-0.08; 009) |
| Rater’s managerial responsibility | -0.04 | 0.04 | -0.98 | (-0.13; 0.04) |
| **Perceived Competence** |  |  |  |  |
|  |  |  |  |  |
| **Predictors** |  |  |  |  |
| Leadership Behavior | 0.31** | 0.07 | 4.58** | (0.17; 0.44) |
| Leader Gender | -0.04 | 0.07 | -0.64 | (-0.18; 0.09) |
| Gender Role Beliefs (median-centered) | 0.07 | 0.11 | 0.67 | (-0.13; 0.30) |
| Leadership behavior*Leader Gender | 0.01 | 0.08 | 0.14 | (-0.14; 0.18) |
| Leadership behavior*Gender Role Beliefs | -0.01 | 0.10 | -0.12 | (-0.22; 0.19) |
| Gender Role Beliefs*Leader Gender | 0.07 | 0.11 | 0.66 | (-0.14; 0.28) |
| Leadership behavior*Leader Gender* Gender Role Beliefs | -0.01 | 0.11 | -0.05 | (-0.22; 0.20) |
| Rater age | -0.09 | 0.04 | -2.00 | (-0.18; -0.00) |
| Rater gender | 0.01 | 0.05 | 0.29 | (-0.08; 0.11) |
| Rater’s managerial responsibility | -0.10 | 0.05 | -2.09 | (-0.20; -0.01) |
| **Perceived Dominance** |  |  |  |  |
| **Predictors** |  |  |  |  |
| Leadership behavior | -0.78** | 0.04 | -17.98** | (-0.86; -0.69) |
| Leader gender | 0.03 | 0.05 | 0.53 | (-0.08; 0.14) |
| Gender Role Beliefs (median-centered) | 0.08 | 0.08 | 0.96 | (-0.09; 0.23) |
| Leadership behavior*Leader gender | 0.01 | 0.06 | 0.21 | (-0.10; 0.13) |
| Leadership behavior*Gender Role Beliefs | -0.20* | 0.08 | -2.52* | (-0.36; -0.04) |
| Gender Role Beliefs*Leader Gender | 0.00 | 0.08 | 0.01 | (-0.16; 0.16) |
| Leadership behavior*Leader Gender* Gender Role Beliefs | 0.01 | 0.08 | 0.13 | (-0.16; 0.17) |
| Rater age | 0.04 | 0.03 | 1.05 | (-0.03; 0.10) |
| Rater gender | -0.02 | 0.03 | -0.53 | (-0.08; 0.05) |
| Rater’s managerial responsibility | 0.04 | 0.03 | 1.04 | (-0.03; 0.10) |
|  |  |  |  |  |
| **Perceived Leader Effectiveness** | |  |  |  |
| **Predictors** |  |  |  |  |
| Leadership behavior | 0.11* | 0.05 | 2.37* | (0.02; 0.21) |
| Perceived Warmth | -0.14 | 0.09 | -1.57 | (-0.30; 0.03) |
| Perceived Morality | 0.13 | 0.10 | 1.26 | (-0.08; 0.33) |
| Perceived Competence | 0.50** | 0.05 | 10.35** | (0.40; 0.60) |
| Perceived Dominance | -0.41** | 0.09 | -4.67** | (-0.57; -0.23) |
| Rater age | -0.02 | 0.03 | -0.74 | (-0.07; 0.04) |
| Rater gender | 0.02 | 0.03 | 0.74 | (-0.04; 0.08) |
| Rater’s managerial responsibility | -0.05 | 0.03 | -1.42 | (-0.11; 0.02) |
|  |  |  |  |  |
| **Perceived Leader Liking** |  |  |  |  |
| **Predictors** |  |  |  |  |
| Leadership behavior | 0.13** | 0.05 | 2.74** | (0.04; 0.22) |
| Perceived Warmth | 0.07 | 0.09 | 0.85 | (-0.09; 0.24) |
| Perceived Morality | 0.19* | 0.08 | 2.50* | (0.04; 0.34) |
| Perceived Competence | 0.24** | 0.05 | 4.55** | (0.14; 0.33) |
| Perceived Dominance | -0.48** | 0.07 | -6.66** | (-0.61; -0.33) |
| Rater age | -0.00 | 0.03 | -0.06 | (-0.05; 0.05) |
| Rater gender | 0.03 | 0.03 | 1.09 | (-0.02; 0.08) |
| Rater’s managerial responsibility | 0.02 | 0.03 | 0.82 | (-0.03; 0.08) |
| **Perceived Leader Promotability** | |  |  |  |
| **Predictors** |  |  |  |  |
| Leadership behavior | 0.07 | 0.05 | 1.54 | (-0.02; 0.16) |
| Leader gender | -0.01 | 0.03 | -0.18 | (-0.07; 0.06) |
| Gender Role Beliefs (median-centered) | -0.06 | 0.05 | -1.16 | (-0.16; 0.04) |
| Leadership behavior*Leader gender | 0.04 | 0.04 | 0.85 | (-0.05; 0.12) |
| Leadership behavior*Gender Role Beliefs | 0.04 | 0.05 | 0.79 | (-0.06; 0.14) |
| Gender Role Beliefs*Leader Gender | 0.04 | 0.05 | 0.65 | (-0.07; 0.14) |
| Leadership behavior* Leader Gender* Gender Role Beliefs | 0.02 | 0.06 | 0.38 | (-0.09; 0.13) |
| Perceived Warmth | -0.12* | 0.06 | -2.07* | (-0.24; 0.00) |
| Perceived Morality | -0.01 | 0.06 | -0.20 | (-0.14; 0.11) |
| Perceived Competence | 0.14** | 0.05 | 2.95** | (0.04; 0.22) |
| Perceived Dominance | -0.15** | 0.05 | -3.07** | (-0.25; -0.06) |
| Perceived Effectiveness | 0.39** | 0.05 | 7.66** | (0.29; 0.49) |
| Perceived Liking | 0.37** | 0.06 | 6.49** | (0.26; 0.48) |
| Rater age | -0.03 | 0.02 | -1.41 | (-0.08; 0.01) |
| Rater gender | -0.01 | 0.03 | -0.29 | (-0.06; 0.04) |
| Rater’s managerial responsibility | -0.01 | 0.02 | -0.47 | (-0.06; 0.03) |
|  |  |  |  |  |

*Note.* **p* < .05; ***p* < .01. Directive leadership is coded as 0, servant leadership as 1. Men are coded as 0, women are coded as 1. Rater’s managerial responsibility, no coded as 0, yes coded as 1.

# Supplementary References

Ames, D. (2009). Pushing up to a point: Assertiveness and effectiveness in leadership and interpersonal dynamics. *Res. Organ. Behav.* 29**,** 111–133. doi: 10.1016/j.riob.2009.06.010

Ames, D. R., and Flynn, F. J. (2007). What breaks a leader: The curvilinear relation between assertiveness and leadership. *J. Pers. Soc. Psychol.* 92**,** 307–324. doi: 10.1037/0022-3514.92.2.307

Jost, J. T., Banaji, M. R., and Nosek, B. A. (2004). A decade of system justification theory: Accumulated evidence of conscious and unconscious bolstering of the status quo. *Pol. Psychol.* 25**,** 881–919. doi: 10.1111/j.1467-9221.2004.00402.x

Khademi, M., Mast, M. S., Zehnder, C., and De Saint Priest, O. (2021). The problem of demand effects in power studies: Moving beyond power priming. *Leadersh. Q.* 32, 101496. doi: 10.1016/j.leaqua.2021.101496

Koenig, A. M., and Eagly, A. H. (2014). Evidence for the social role theory of stereotype content: Observations of groups’ roles shape stereotypes. *J. Pers. Soc. Psychol.* 107**,** 371–392. doi: 10.1037/a0037215

Kray, L. J., Howland, L., Russell, A. G., and Jackman, L. M. (2017). The effects of implicit gender role theories on gender system justification: Fixed beliefs strengthen masculinity to preserve the status quo. *J. Pers. Soc. Psychol.* 112**,** 98–115. doi: 10.1037/pspp0000124

Muthén, L. K., and Muthén, B. O. (1998–2017). *Mplus User’s Guide*, 8^th^ Edn. Los Angeles, CA: Muthén & Muthén.

Zehnter, M. K., Manzi, F., Shrout, P. E., and Heilman, M. E. (2021). Belief in sexism shift: Defining a new form of contemporary sexism and introducing the belief in sexism shift scale (BSS scale). *PLoS ONE* 16, e0248374. doi: 10.1371/journal.pone.0248374

1. We also examined the gendered leadership expectations for transformational leadership in the first pre-study to check whether our design produced the same results as Hentschel et al. (2018) which it did. [↑](#footnote-ref-1)
2. The perceived age of the leader was not normally distributed in the conditions directive woman leader (*p* < .01), servant woman leader, and directive man leader (*p* < .05) as assessed by the Shapiro-Wilk test. Outliers per condition remained in the analyses as results did not differ when they were excluded. [↑](#footnote-ref-2)
3. We squared the values for the moderator raters’ gender role beliefs and used the squared data as moderator in the analyses due to the left skewed nature of the variable and its lack of linearity discovered by visual inspection of the scatterplots after LOESS smoothing. We included the original values for gender role beliefs as additional covariate. [↑](#footnote-ref-3)
